# The metabolic recovery of marathon runners: an untargeted ^1^H-NMR metabolomics perspective

**DOI:** 10.3389/fphys.2023.1117687

**Published:** 2023-05-04

**Authors:** Rachelle Bester, Zinandré Stander, Shayne Mason, Karen M. Keane, Glyn Howatson, Tom Clifford, Emma J. Stevenson, Du Toit Loots

**Affiliations:** ^1^ Human Metabolomics, Department of Biochemistry, Faculty of Natural and Agricultural Sciences, North-West University, Potchefstroom, South Africa; ^2^ Department of Sport Exercise and Nutrition, School of Science and Computing, Atlantic Technological University, Galway, Ireland; ^3^ Department of Sport, Exercise and Rehabilitation, Faculty of Health and Life Sciences, Northumbria University, Newcastle upon Tyne, United Kingdom; ^4^ Water Research Group, School of Environmental Sciences and Development, North-West University, Potchefstroom, South Africa; ^5^ School of Sport, Exercise and Health Sciences, Loughborough University, Loughborough, United Kingdom; ^6^ Human and Exercise Nutrition Research Centre, School of Biomedical, Nutritional and Sport Sciences, Faculty of Medical Sciences, Newcastle University, Newcastle upon Tyne, United Kingdom

**Keywords:** metabolomics, metabolism, marathon, 1H-NMR spectroscopy, recovery, endurance running, delayed onset muscle soreness (DOMS)

## Abstract

**Introduction:** Extreme endurance events may result in numerous adverse metabolic, immunologic, and physiological perturbations that may diminish athletic performance and adversely affect the overall health status of an athlete, especially in the absence of sufficient recovery. A comprehensive understanding of the post-marathon recovering metabolome, may aid in the identification of new biomarkers associated with marathon-induced stress, recovery, and adaptation, which can facilitate the development of improved training and recovery programs and personalized monitoring of athletic health/recovery/performance. Nevertheless, an untargeted, multi-disciplinary elucidation of the complex underlying biochemical mechanisms involved in recovery after such an endurance event is yet to be demonstrated.

**Methods:** This investigation employed an untargeted proton nuclear magnetic resonance metabolomics approach to characterize the post-marathon recovering metabolome by systematically comparing the pre-, immediately post, 24, and 48 h post-marathon serum metabolite profiles of 15 athletes.

**Results and Discussion:** A total of 26 metabolites were identified to fluctuate significantly among post-marathon and recovery time points and were mainly attributed to the recovery of adenosine triphosphate, redox balance and glycogen stores, amino acid oxidation, changes to gut microbiota, and energy drink consumption during the post-marathon recovery phase. Additionally, metabolites associated with delayed-onset muscle soreness were observed; however, the mechanisms underlying this commonly reported phenomenon remain to be elucidated. Although complete metabolic recovery of the energy-producing pathways and fuel substrate stores was attained within the 48 h recovery period, several metabolites remained perturbed throughout the 48 h recovery period and/or fluctuated again following their initial recovery to pre-marathon-related levels.

## 1 Introduction

The successful completion of endurance running events, such as half-marathons (21.1 km), marathons (42.2 km), and ultra-marathons (>42.2 km) (Ambrozic et al., 2018), is regarded as a remarkable physical (Stöggl and Wunsch, 2016) and mental accomplishment (Hammer and Podlog, 2016). Whether participating recreationally or competitively, these events result in considerable physiological (Sperlich, 2016; D’Silva et al., 2020) and immunological (Nieman, 2007; Barros et al., 2017) responses, in addition to triggering acute metabolic shifts in various energy-producing pathways (Lewis et al., 2010; Schader et al., 2020). These pathways can work in concert with each other, and the relative contribution of each pathway may vary depending on the intensity and duration of the exercise. During a marathon, these pathways may work simultaneously to varying degrees, depending on factors such as the runner’s fitness level, and training and nutrition status. Energy production during endurance exercise typically involves upregulation in both anaerobic and aerobic glycolysis followed by the activation of fatty acid oxidation, amino acid catabolism, ketogenesis, and tricarboxylic acid (TCA)-cycle activity (Stander et al., 2018; Bester et al., 2021), when the glycogen stores or glucose supplies/uptake becomes inadequate (Schader et al., 2020). Furthermore, the exhaustive nature of a marathon is emphasized by the eventual use of other minor energy-producing pathways, including ω-oxidation of fatty acids (Nieman et al., 2013) and autophagy as a means to supply the necessary energy required for completing such an extreme running event (Stander et al., 2018; Dalle Carbonare et al., 2019). Based on the magnitude of these perturbations, it is thought that frequent participation in exhaustive exercises accompanied by inadequate recovery may result in functional overreaching and overtraining syndrome (Nieman and Mitmesser, 2017). In contrast, adequate post-exercise recovery reportedly improves athletic performance (Kellmann et al., 2018), in part, via metabolic acclimation in the form of increased mitochondrial density and oxidative function (Egan and Zierath, 2013), increased monocarboxylate transporters (Philippou et al., 2019), and a slower utilization of carbohydrates as fuel substrates coupled with an upregulated lipid metabolism during the running event (Hawley and Leckey, 2015). In an attempt to facilitate/improve the systematic recovery process, a considerable amount of research has focused on investigating the efficacy of a variety of recovery aids (Saunders et al., 2018; Wilson et al., 2018; Kwiecien et al., 2021; Martinez-Navarro et al., 2021). Regardless, to effectively investigate/develop recovery-enhancing modalities, the connatural recovery (i.e., unaided by recovery modalities) of the perturbed metabolome must first be elucidated. To date, recovery following endurance running has mainly been determined by characterizing the pathophysiology of muscle damage/fatigue, cardiovascular strain, and immunological responses to endurance events (Saunders et al., 2018; Wilson et al., 2018; Bernat-Adell et al., 2021; Kwiecien et al., 2021; Martinez-Navarro et al., 2021). Commonly used biological indicators utilized in the aforementioned investigations included creatine kinase, high sensitivity C-reactive protein, troponin T, interleukin-6, lactate dehydrogenase, tumor necrosis factor-alpha, aspartate aminotransferase, and a variety of physiological performance/subjective pain perception indicators including countermovement jumps, perceived muscle soreness, maximum isometric voluntary contractions, reactive strength index, and maximal aerobic capacity (VO_2max_). In summary, perceived muscle soreness, strength, and countermovement jumps typically recovered within 48–72 h (Clifford et al., 2017; Wilson et al., 2018; Kwiecien et al., 2021). Moreover, cardiac and muscle damage markers (creatine kinase, troponin T) returned to baseline levels within 96–144 h, whereas markers associated with liver damage (aspartate aminotransferase) remained perturbed at 48 h post-marathon (Clifford et al., 2017; Bernat-Adell et al., 2021; Martinez-Navarro et al., 2021). This further holds true for lactate dehydrogenase and the inflammatory marker C-reactive protein, which recovered after 192 h; however, interleukin-6 and tumor necrosis factor-alpha recovered within 48 and 24 h, respectively (Clifford et al., 2017). Considering exhaustive exercise, metabolic recovery is thought to proceed immediately following cessation of the exercise bout and mainly involves rapid lactate clearance and the restoration of myocellular oxygen levels, ATP, and phosphocreatine stores. In the subsequent phase, anabolic processes associated with the replenishment of fuel substrate reserves, particularly muscle and hepatic glycogen stores, are upregulated. Furthermore, muscle protein anabolism is also upregulated, resulting in the repair of exercise-induced muscle damage and eventual restoration and/or increase in muscle size and strength (Moore, 2019). It is further indicated that lipid catabolism is upregulated within the first 14 h following a marathon (Lewis et al., 2010; Nieman et al., 2013) and may likely be ascribed to lipids serving as the main energy source during recovery in order to facilitate rapid protein turnover and carbohydrate store restoration. Although these measurements are well described and have been applied with relative success, limited literature is available regarding the underlying biochemical mechanisms associated with the recovery of athletes (Nieman et al., 2013; Stander et al., 2020). Studying the post-marathon recovering metabolome using an untargeted metabolomics approach can aid in the identification of biomarkers that may lead to an improved understanding of the mechanisms related with marathon-induced stress, recovery, and adaptation. This may, in turn, facilitate the development of improved personalized training and recovery programs, and the identification of various metabolite markers that may be applied to monitor of athletic health/recovery.

Metabolomics may be applied for hypothesis-driven (targeted or semi-targeted) or hypothesis-generating (untargeted) research. If an *a priori* established list of specific metabolites or a metabolite class is of interest, a targeted or semi-targeted metabolomics approach would be chosen (Manier and Meyer, 2020). However, if a study is explorative in nature, all metabolites present in a sample are of potential interest; therefore, an untargeted metabolomics approach would be used, wherein the number of metabolites investigated is limited only by the capabilities of the chosen analytical platform and associated sample preparation. Considering this, metabolomics ultimately strives to comprehensively detect, identify, and quantify all endogenous and exogenous metabolites (≤1,500 Da) in a biological system at a specific point in time in the presence/absence of a perturbation (e.g., environmental factors, disease, dietary, and lifestyle changes) (Dunn and Ellis, 2005), which is collectively referred to as the metabolome. The metabolome is an amplified, downstream representation of genomic, transcriptomic, and proteomic responses to genetic, environmental, and physiological factors, thereby providing a closer representation of the altered physical state. Although there are a number of studies that have investigated the recovering metabolism after physical activity, very few have utilized untargeted metabolomics to elucidate the recovery of the metabolome after the completion of an endurance running event involving 3–5 h of continuous running (i.e., a marathon) (Nieman et al., 2013; Stander et al., 2020). This distinguishing factor is a key component as it has been established that different exercise intensities (resistance/endurance/aerobic/anaerobic) and durations (time) evoke different metabolic responses and, therefore, different recovery-based changes (Sakaguchi et al., 2019). While several studies have employed an untargeted metabolomics approach to elucidate the metabolic recovery trend of athletes after endurance running races, these studies mainly used LC-MS and GC-MS directed approaches for metabolite profiling. Although these platforms are highly sensitive, their reproducibility and quantitative abilities are negatively affected by factors such as complex sample preparation, instrument variability, and data processing techniques. Different analytical platforms are suitable to analyze different metabolite classes, hence, obtaining a more comprehensive view of the overall metabolome necessitates the consideration of information generated using a variety of analytical platforms.

^1^H-NMR spectroscopy, as an untargeted metabolomics platform, is not biased to a particular class of compound and provides absolute quantification of identified metabolite markers. Therefore, in addition to potentially identifying metabolites that have not yet been associated with the connatural metabolic recovery of athletes following a marathon, previous hypothesized recovery trends identified using other highly sensitive analytical platforms may also be confirmed. As such, the current investigation employed an untargeted ^1^H-NMR metabolomics approach to analyze serum samples of 15 marathon participants obtained before, immediately, ∼24 h, and ∼48 h after a marathon, to further investigate the connatural metabolic recovery trend of athletes in response to a marathon.

## 2 Materials and methods

**FIGURE 1:**
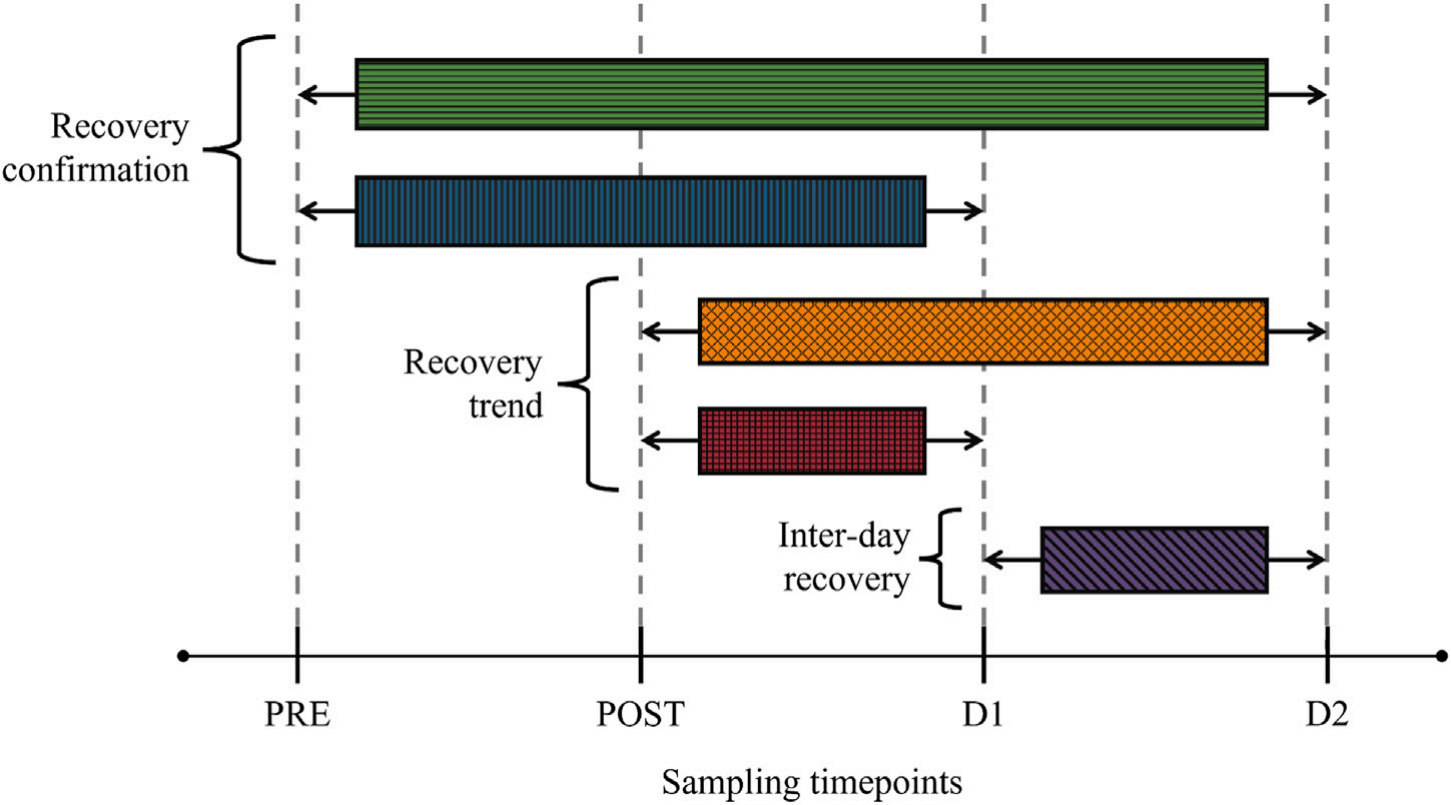
Study design visualizing comparative groups and the sampling timepoints. Recovery confirmation—involved comparing the baseline (PRE) metabolome with corresponding 24 h (D1) and 48 h (D2) post-marathon metabolomes. Recovery trend—involved comparing the perturbed (POST) metabolome with corresponding D1 and D2 metabolomes. Inter-day recovery—involved comparing D1 and D2 metabolomes.

This investigation is an integrant of a collaboration consisting of several inter-disciplinary aims that fall outside the scope of the current investigation, including clinical and physiological post-marathon recovery measurements (Clifford et al., 2017) as well as complementary multi-platform-based metabolomics investigations (Stander et al., 2018; Stander et al., 2020; Bester et al., 2021). As such, participant recruitment/selection, environmental conditions on the day of the marathon, sample handling, and analytical methodologies employed have previously been described (Clifford et al., 2017; Stander et al., 2018; Stander et al., 2020; Bester et al., 2021) and will only be summarized in the sections below. The study design of the current investigation is visually summarized in Figure 1.

### 2.1 Participants

Voluntary participants (*n* = 15; 9 males and 6 females) competing in the Druridge Bay Marathon (Northumberland, United Kingdom) were included in this investigation after providing informed consent. Similar to the current investigation, Stander et al. (2020) only characterized the relevant subsection (*n* = 16) of the complete participant group (*n* = 34). However, due to limited sample volume, one participant was excluded from the current investigation (*n* = 15). Eligibility assessment (Clifford et al., 2017) was conducted by means of a health screening questionnaire whereafter those individuals with food hypersensitivity, cardiovascular and musculoskeletal complications/diseases, or those undergoing anti-inflammatory treatment were excluded from the investigation. Female participants were also required to complete a menstrual cycle questionnaire to identify potential hormonal influences. Furthermore, all participating athletes were required to refrain from using any putative recovery aids (e.g., ice baths and compression garments) during the course of this investigation. A summary of participant demographics is provided in Table 1. Ethical clearance was obtained from both the Research Ethics Committee of the Faculty of Health and Life Sciences at the Northumbria University in Newcastle, United Kingdom (HLSTC120716) and the Health Research Ethics Committee of the North-West University, South Africa (NWU-00163-21-A1), as conducted in accordance with the Declaration of Helsinki and International Conference on Harmonization Guidelines.

**TABLE 1:** Participant demographics.

Participant characteristics	Average ± standard deviation
Age (years)	40 ± 13
Gender (male/female)	9/6
Pre-marathon mass (kg)	71 ± 10
Post-marathon mass (kg)	69 ± 10
Weekly training distance (km)	53 ± 18
Running experience (years)	8 ± 7
Track run record (km)	34 ± 4
Marathon finishing time (hh:mm:ss)	04:25:18 ± 00:53:29
Running velocity (km/h)	10 ± 2

### 2.2 Environmental conditions

The 2016 Druridge Bay marathon (Northumberland coast) consisted of four-laps in and around the area of the Druridge Bay country park (Morpeth, United Kingdom). The route was level and encompassed a combination of paved, grassy, and soft sand terrain, the latter covering approximately 6.4 km of the total marathon distance. Environmental conditions were recorded at the commencement (09:00) and conclusion (approximately 13:30) of the marathon (Table 2). Weather conditions remained mostly cloudy with occasional sunshine throughout the race (Clifford et al., 2017).

**TABLE 2:** Marathon environmental factors.

	Marathon start time	Marathon finishing time
Ambient temperature (°c)	3.8	8.5
Wind speed (km/h)	9	14
Humidity (%)	82	62
Barometric pressure (hpa)	1013	1013

### 2.3 Sample collection and storage

Blood samples were collected via antecubital fossa venesection of the basilica vein into 10 mL serum separator tubes before (PRE), immediately after (POST), 24 h (D1), and 48 h (D2) after the successful completion of the marathon. As part of the initial sample processing steps, samples were allowed to coagulate for 30 min before being centrifuged. All serum samples were frozen at −80°C and transported on dry ice to the North-West University, Human Metabolomics: Laboratory of Infectious and Acquired Diseases, South Africa. Here, the serum samples were stored at −80°C prior to metabolomics analyses. Further details regarding sample collection and storage are provided by Stander et al., 2020 and Bester et al., 2021.

### 2.4 Miniaturized ^1^H-NMR sample preparation

All samples were randomized and divided into five batches prior to sample preparation and analyses. A pooled quality control (QC) sample, containing 50 µL of each test sample, was prepared and subsequently aliquoted (three per batch, *n* = 15) in order to avoid multiple freeze/thaw cycles. The buffer solution and samples were prepared by means of a miniaturized ^1^H-NMR method adapted from Mason et al. (2018) and described in detail by Bester et al. (2021). Briefly, serum macromolecules were removed by filtering 100 µL of each test and QC sample at 6,000 × *g* for 20 min using pre-rinsed (three times) centrifugal filters (Centrifree Ultacel membrane filters, Merck Millipore, Carrigtwohill, Ireland; 10,000 Da pore size), subsequently avoiding the occurrence of spectral interference and poor spectral baseline caused by proteins. An eVol^®^ NMR automated digital syringe system (Supelco, St. Louis, United States) with a pre-programmed pipetting sequence was utilized to dispense 54 µL of the filtrate and 6 µL buffer solution into 2 mm ^1^H-NMR tubes. All samples were then loaded onto a SampleXpress autosampler utilizing the Bruker MATCH system. QC samples were strategically analyzed at the beginning, middle, and end of each batch in order to monitor analytical repeatability throughout the entire analysis.

### 2.5 ^1^H-NMR spectroscopic analyses

All participant and QC samples were analyzed as described by Bester et al. (2021) using a Bruker Avance III HD 500 MHz NMR spectrometer, equipped with a 5 mm triple-resonance inverse (TXI) probe head. To summarize, the experimental parameters of each sample being analyzed were adjusted using Bruker Topspin software (version 3.5) and included shimming to the trimethylsilylpropanoic acid (TSP, internal standard) signal, automatic signal locking to D_2_O, tuning of the probe head, and pulse calibration. An excitation pulse of 90° was applied to each scan (*n* = 128) for 8 μs, followed by a relaxation delay of 4 s. Finally, the ^1^H-NMR spectral width was 6,000 Hz (12.0 ppm).

### 2.6 Data processing and clean-up

Prior to data processing, Bruker Topspin software (version 3.5) was used to perform data pre-processing steps, namely, Fourier-transformation, baseline phasing/correction, TSP peak calibration, and H_2_O resonance suppression using the NOESY-presat pulse sequence program. Additionally, manual inspection of the TSP peak width (<1 Hz at half height) was done to ensure proper shimming was applied. Bruker AMIX software (version 3.9.14) was utilized for further data processing, which involved normalization relative to TSP and variable-sized binning (132 bins). In contrast to fixed-sized binning, where each bin has a uniform width, variable-sized binning involves the adjustment of the size of the bins to facilitate greater flexibility in capturing spectral features that may vary in intensity and/or chemical shift, thereby avoiding peaks being split across multiple bins and removing regions of spectral noise, subsequently eliminating the influence of spectral noise during statistical analyses. Data clean-up was executed using MetaboAnalyst version 5.0 (Pang et al., 2021), and included heteroscedasticity correction via natural shift log transformation and auto-scaling.

### 2.7 Statistical analyses

**FIGURE 2:**
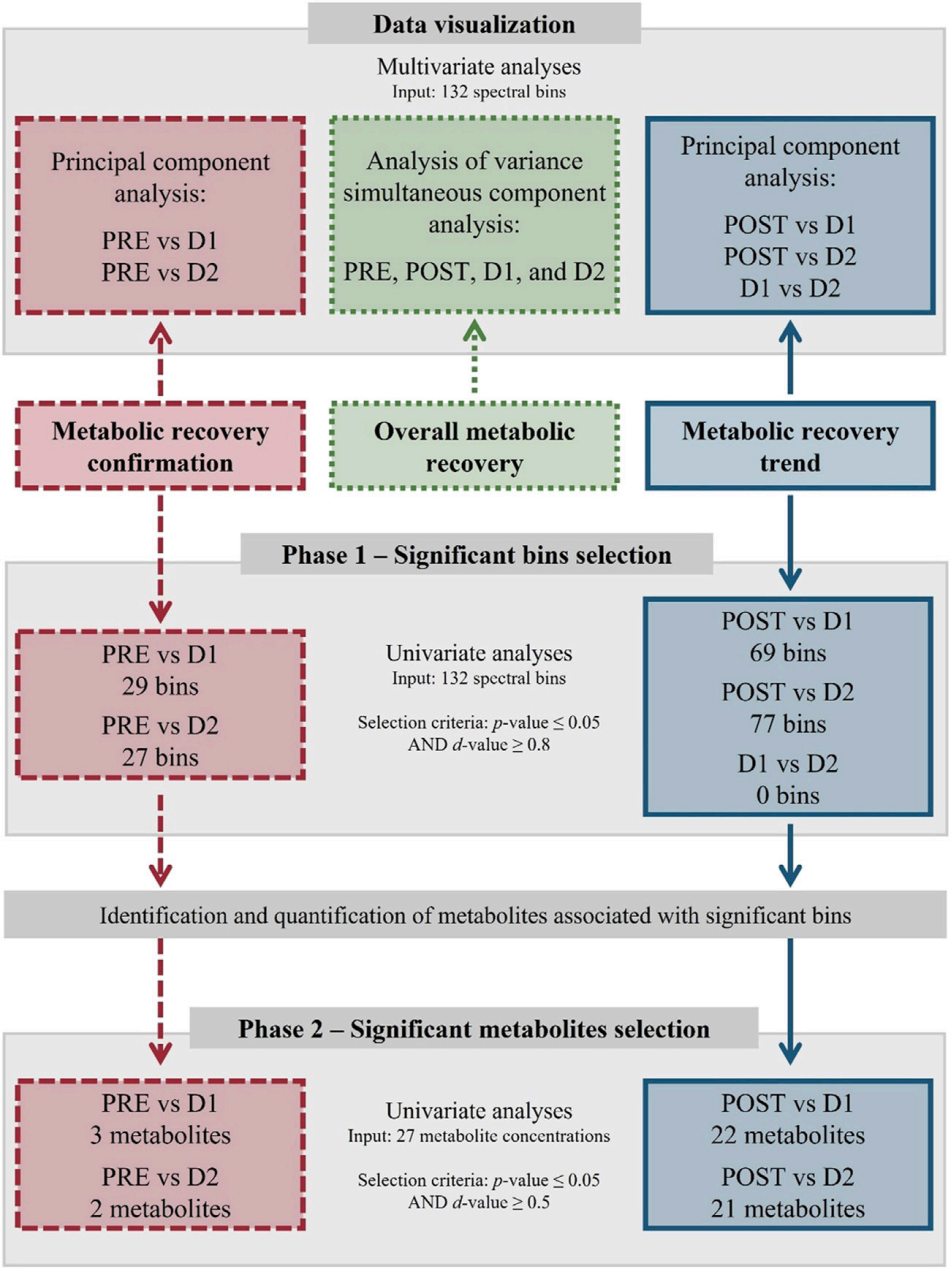
Multi-statistical approach summary. Statistical design indicating data visualization, significant bins selection, and significant metabolites selection methods/criteria. Comparative groups include baseline (PRE), perturbed (POST), 24 h (D1), and 48 h (D2) post-marathon, and are shown in relation to the three statistical objectives: metabolic recovery confirmation (red, dashed lines), overall metabolic recovery visualization (green, dotted lines), and metabolic recovery trend characterization (blue, solid lines).

Firstly, in order to obtain a global view of the metabolic changes over the entire study timeframe, binned data of all the timepoints (PRE, POST, D1, and D2) were subjected to an analysis of variance (ANOVA) simultaneous component analysis (ASCA) (Smilde et al., 2005; Zwanenburg et al., 2011) using MATLAB version R2021b (Moler, 2021) equipped with the PLS Toolbox version 9.0 (Eigenvector Research, 2021). Secondly, comprehensive uni- and multivariate pairwise comparisons were used to identify those metabolites that 1) did not recover within 24 h (PRE vs. D1) and 48 h (PRE vs. D2) after the marathon, and 2) fluctuated significantly during the 24 h (POST vs. D1) and 48 h (POST vs. D2 and D1 vs. D2) recovery periods. A schematic representation of the statistical group comparisons and the multi-statistical approach used are provided in Figures 1, 2, respectively. Multivariate analyses were performed to visually evaluate the variances between each comparative group and included principal component analyses (PCA) using MetaboAnalyst (version 5.0). Thereafter, univariate analyses of the pairwise comparisons transpired in a bi-phasic manner to identify significant bins (phase 1) and subsequently select significant metabolites (phase 2) based on the results from phase 1. These univariate analyses included a paired t-test corrected for multiple testing by the Benjamini-Hochberg procedure (MetaboAnalyst, version 5.0), as well as an independent effect size calculation (Excel 2019; Microsoft 365, version 2204), based on Glass’s Δ effect size, as described by Ialongo. (2016). During phase 1, statistical significance was determined by means of an adjusted *p*-value (≤0.05) and a large effect size (*d*-value ≥ 0.8), whereafter the associated peaks of the selected bins were identified and quantified (Section 2.8). During phase 2, metabolites were selected based on an adjusted *p*-value (≤0.05) and a moderate effect size (*d*-value ≥ 0.5). Statistically significant metabolites were then subjected to quantitative enrichment analyses (MetaboAnalyst, version 5.0) using the Kyoto Encyclopedia of Genes and Genomes (KEGG; Supplementary Figure S1) and Small Molecule Pathway Database (SMPDB; Supplementary Figure S2) as reference, thereby aiding in their interpretation. Finally, a metabolic map visualizing the main metabolic pathways involved in the identified metabolite fluctuations was constructed using PowerPoint 2016 (Microsoft 365, version 2204).

### 2.8 Metabolite identification, confirmation, and absolute quantification

Metabolites were identified using a pure chemical compound library, of which the respective ^1^H-NMR assignments are indicated in Supplementary Table S1. To establish a level 1 confidence identification (Schrimpe-Rutledge et al., 2016), metabolite identities were confirmed using homonuclear-correlation spectroscopy (COSY) and homonuclear J-resolved spectroscopy (JRES) 2D-NMR analyses (Bester et al., 2021). COSY and JRES spectra were acquired while maintaining identical experimental conditions to that of the 1D-^1^H-NMR analyses, allowing for accurate correlations between 1D and 2D data of non-novel metabolites. A spectral width of 8,000 Hz per dimension was recorded for both COSY and JRES spectra, at 16 scans per increment, 2 s recycle delay, and 8.5 µs pulse. ^1^H-NMR based quantification of the identified metabolite concentrations was done by associating the number of protons represented by those metabolite peaks with a good signal-to-noise ratio to their signal intensities. Thereafter, the data were equated to the TSP signal concentration (0.5805 mM), which is produced by a known number of protons (H^+^ = 9). Considering that quantification is based on the exact number of protons of each compound, it is possible to calculate the absolute concentration of every detected metabolite.

## 3 Results

The multi-statistical approach discussed in Section 2.7 (Figure 2) involved the use of multivariate analyses to visualize the metabolomics data, and univariate analyses to identify significant metabolites. Following phase 1 of the statistical analyses, 29 (PRE vs. D1), 27 (PRE vs. D2), 69 (POST vs. D1), 77 (POST vs. D2), and 0 (D1 vs. D2) bins of the initial 132 bins were identified to vary significantly (*p*-value ≤ 0.05 and *d*-value ≥ 0.8) in-between timepoints. Following phase 2, 3 (PRE vs. D1; Supplementary Table S2), 2 (PRE vs. D2; Supplementary Table S3), 22 (POST vs. D1; Supplementary Table S4), 22 (POST vs. D2; Supplementary Table S5), and 0 (D1 vs. D2) statistically significant (*p*-value ≤ 0.05 and *d*-value ≥ 0.5) metabolites were selected. In total, 26 distinct metabolites were obtained, the concentrations of which are presented in Table 3. The metabolic impact of the marathon itself (PRE vs. POST) has previously been investigated and may be referred to for further information (Stander et al., 2018; Bester et al., 2021). The results below commences with 1) an overall evaluation of the recovering metabolome (PRE, POST, D1, and D2; Section 3.1), followed by 2) the identification those metabolites that did not recover (PRE vs. D1 and PRE vs. D2; Section 3.2), and 3) those that changed significantly (POST vs. D1 and POST vs. D2; Section 3.3) within the 24 and 48 h recovery periods.

**TABLE 3:** Metabolites associated with post-marathon recovery—Average concentrations (and standard deviation) of all 26 statistically significant serum metabolites that changed before (PRE), directly after (POST), 24 h (D1), and 48 h (D2) after completion of a marathon.

Metabolite (PubChem ID)	Average concentration in ΜM (standard deviation)			
	PRE	POST	D1	D2
2-hydroxybutyric acid (11266)^c,d^	34.2 (15.7)	62.4 (17.5)	35.2 (15.7)	34.2 (13.6)
3-hydroxybutyric acid (441)^c,d^	53.4 (32.8)	448.0 (271.1)	43.6 (13.7)	46.9 (18.0)
3-hydroxyisobutyric acid (87)^c,d^	19.5 (6.2)	37.3 (10.3)	17.8 (6.2)	18.1 (7.0)
3-methyl-2-oxovaleric acid (47)^c^	9.4 (3.8)	11.8 (3.9)	8.2 (3.6)	8.8 (3.5)
Acetoacetic acid (96)^c,d^	19.9 (6.4)	53.6 (25.9)	18.6 (4.7)	18.5 (4.4)
Acetone (180)^c,d^	7.0 (2.4)	17.1 (11.6)	7.7 (2.5)	7.2 (1.7)
Acetylcarnitine (7045767)^c,d^	9.1 (3.3)	26.8 (8.6)	8.7 (2.1)	9.0 (3.2)
Citric acid (311)^c,d^	136.6 (23.5)	209.0 (60.6)	128.4 (18.4)	123.0 (29.2)
Creatine (586)^c,d^	63.1 (13.8)	90.4 (27.4)	63.2 (16.1)	71.1 (21.8)
Creatinine (588)1^c,d^	49.5 (10.2)	65.7 (16.4)	49.9 (8.4)	53.1 (7.9)
Ethanol (702)^a,b,c,d^	30.4 (7.3)	54.2 (55.2)	14.8 (6.3)	16.7 (6.5)
Glucose (5793)^c^	1518.5 (387.2)	1866.7 (459.6)	1393.0 (256.2)	1498.0 (310.2)
Hypoxanthine (135398638)^c^	9.6 (4.6)	13.7 (6.5)	6.9 (4.0)	7.0 (3.6)
Isoleucine (6306)^c,d^	69.0 (23.0)	49.7 (13.6)	62.5 (15.2)	64.8 (19.7)
Lactic acid (612)^c,d^	2506.9 (750.5)	4577.1 (1354.3)	2467.0 (490.5)	2528.0 (711.1)
Leucine (6106)^c,d^	159.3 (43.2)	119.5 (25.3)	136.0 (27.4)	140.8 (32.9)
Lysine (5962)^c,d^	171.5 (52.0)	127.7 (26.4)	157.3 (32.5)	173.2 (33.8)
Methanol (887)^a^	79.5 (19.3)	63.3 (14.4)	65.7 (16.6)	70.7 (12.4)
Ornithine (389)^d^	107.4 (27.4)	82.2 (20.9)	101.2 (25.3)	113.4 (29.1)
Phenylalanine (6140)^d^	102.9 (31.4)	121.8 (50.2)	93.9 (37.7)	75.0 (30.5)
Proline (145742)^c,d^	314.4 (80.7)	220.8 (62.4)	292.9 (49.6)	284.1 (57.1)
Propylene glycol (1030)^c,d^	13.8 (12.5)	18.0 (8.2)	11.2 (5.3)	11.8 (8.6)
Pyruvic acid (1060)^c,d^	60.3 (23.1)	112.3 (40.9)	68.4 (25.1)	60.1 (22.4)
Succinic acid (1110)^c,d^	15.8 (2.5)	24.2 (7.5)	14.4 (2.2)	16.0 (3.0)
Tyrosine (6057)^b,d^	69.3 (15.5)	76.2 (21.2)	67.3 (19.5)	53.3 (18.3)
Valine (6287)^a,c,d^	262.7 (65.2)	198.0 (38.2)	225.0 (42.5)	236.9 (52.2)

Statistical significance between comparative groups (^a^PRE vs. D1, ^b^PRE vs. D2, ^c^POST vs. D1, and, ^d^POST vs. D2) based on *p*-value < 0.05 and *d*-value ≥ 0.5.

### 3.1 The overall metabolic recovery within 48 h post-marathon

**FIGURE 3:**
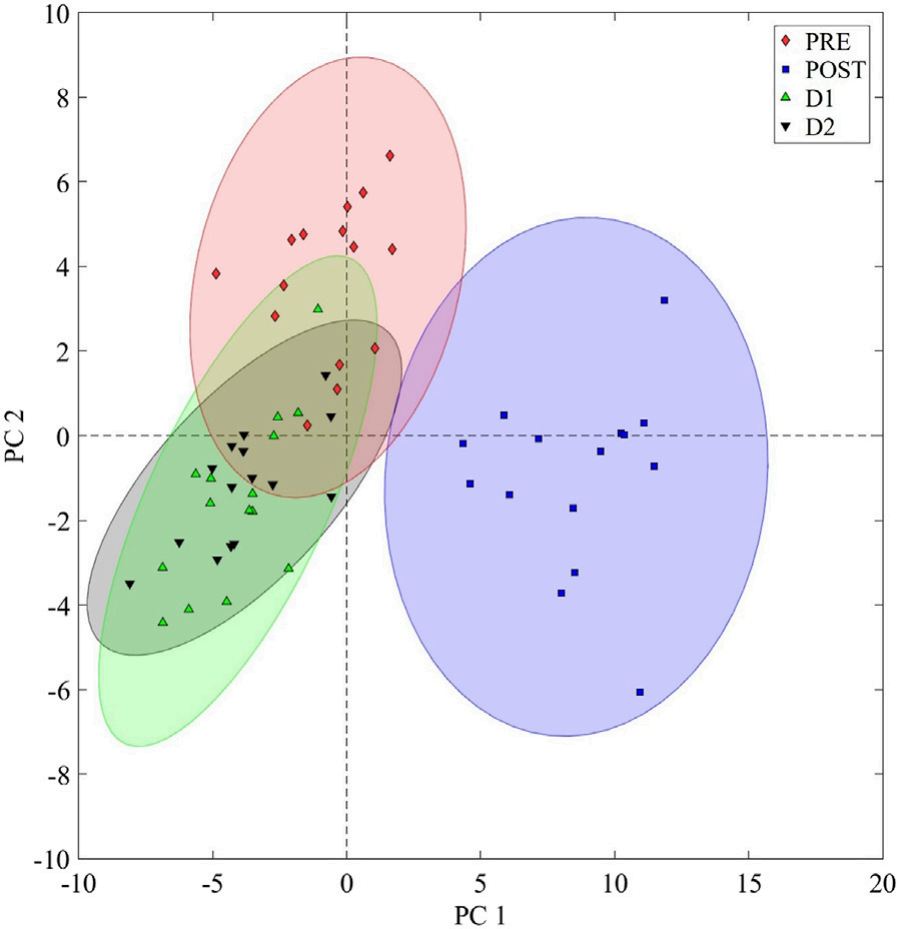
General metabolome recovery trend. An analysis of variance (ANOVA) simultaneous component analysis (ASCA) plot visualizing the time dependent recovery of the marathon-induced perturbed metabolome. PRE: Pre-marathon; POST: directly post-marathon; D1: 24 h post-marathon; D2: 48 h post-marathon; PC: Principal component. A 95% confidence interval is displayed.

To visualize the overall (PRE, POST, D1, and D2) metabolic changes of the runners, an ASCA model was constructed, and the resulting scores were grouped according to the time component of the current study design (Figure 3). Evidently, a nearly complete metabolic recovery to a pre-marathon-related state was achieved within 48 h, as indicated by the relative propinquity of the D1 and D2 ellipsoids to that of PRE. However, the slight off-center positioning of these ellipsoids may suggest the presence of small differences between the pre-marathon and recovering metabolomes. Subsequent pairwise comparisons were therefore performed to identify these differences.

### 3.2 Confirmation of metabolome recovery to a pre-marathon related state

**FIGURE 4:**
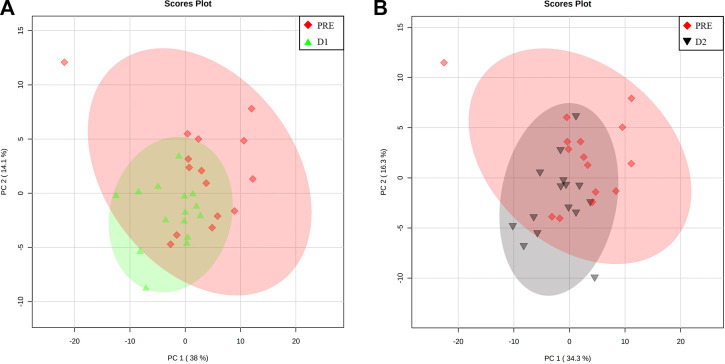
Metabolome recovery confirmation. Principal component (PC) analysis plots indicating baseline (PRE, red diamonds) metabolome data relative to that of the **(A)** 24 h (D1, green triangles) and **(B)** 48 h (D2, black upside-down triangle) post-marathon metabolomes. A 95% confidence interval is displayed.

Pairwise multivariate analyses commenced with the comparison of the PRE metabolite profiles of athletes to corresponding D1 and D2 profiles, as a means of determining whether the serum metabolome of the athletes has indeed recovered to the pre-marathon state. When considering the PCA plots of the respective pairwise comparisons (Figures 4A, B), the confined positioning of the D1 and D2 ellipsoids relative to that of PRE confirms metabolic recovery of athletes within 48 h post marathon. However, univariate statistical methods indicated that 3 (valine, ethanol, and methanol; Supplementary Table S2; Supplementary Figure S3) and 2 metabolites (ethanol and tyrosine; Supplementary Table S3; Supplementary Figure S4) remained perturbed (*p*-value ≤ 0.05 and *d*-value ≥ 0.5) after 24 and 48 h, respectively. The concentration changes of these metabolites are presented in Table 3.

### 3.3 Significant metabolite changes during post-marathon recovery

**FIGURE 5:**
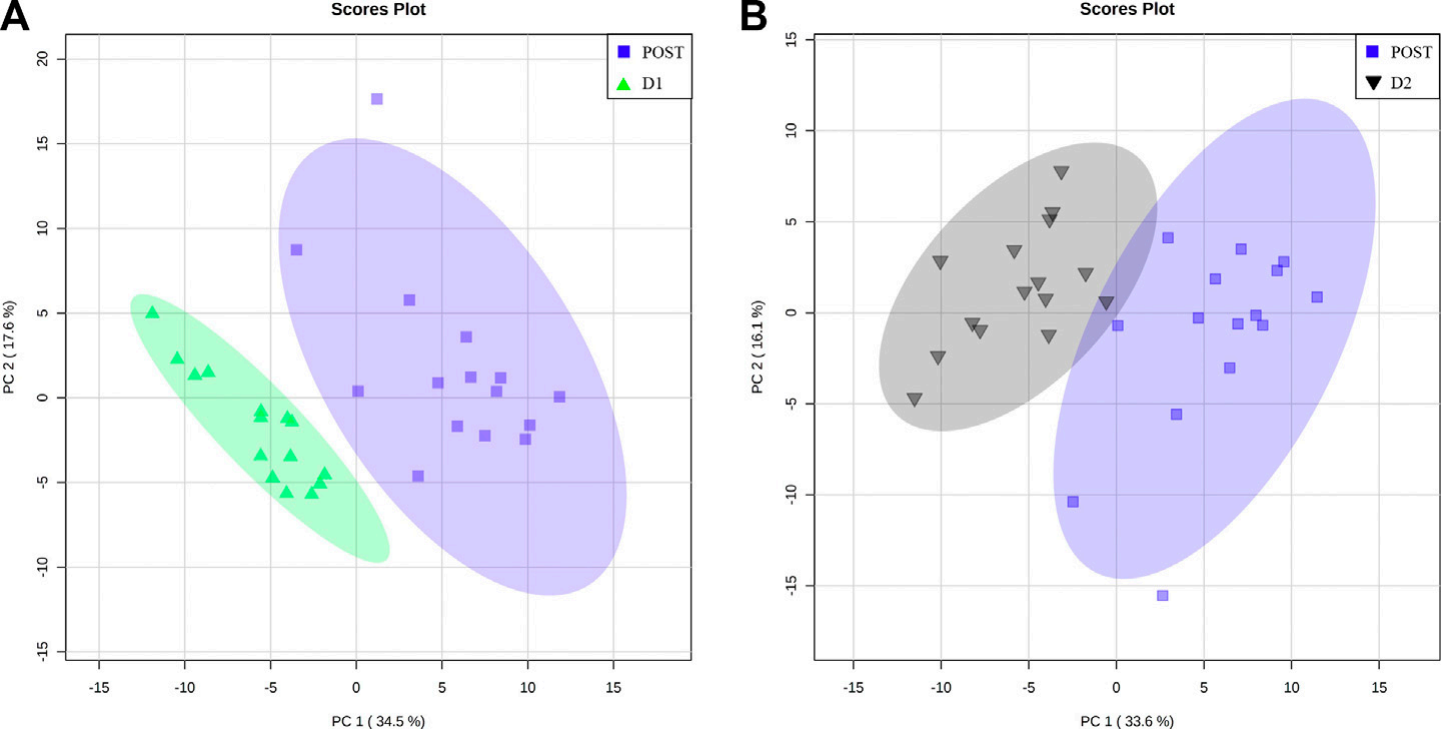
Metabolome recovery trend. Principal component (PC) analysis plots indicating perturbed (POST, blue square) metabolome data relative to that of the **(A)** 24 h (D1, green triangles) and **(B)** 48 h (D2, black upside-down triangle) post-marathon metabolomes. A 95% confidence interval is displayed.

To further elucidate the recovery trend of the perturbed metabolome within 24 and 48 h post-marathon, the POST metabolite profiles of the athletes were compared to their corresponding D1 and D2 profiles. The respective PCA plots of these comparisons (Figures 5A, B) demonstrate the extent to which the metabolome changes relative to the perturbed POST state during the recovery period. Concentrations of the metabolites (*n* = 22) that appear to be significantly affected (*p*-value ≤ 0.05 and *d*-value ≥ 0.5) during the first 24 h (Supplementary Table S4) and 48 h (Supplementary Table S5) of recovery are provided in Table 3.

## 4 Discussion

To determine which mechanisms are pertinent to the metabolic recovery of athletes after a marathon, it is imperative to first consider the metabolic changes induced by this endurance event. Although this falls outside of the scope of the current investigation, these changes have previously been characterized (Lewis et al., 2010; Stander et al., 2018; Hargreaves and Spriet, 2020; Bester et al., 2021), and may be referred to for a comprehensive overview. Briefly, previous literature reports adaptations in fuel substrate and associated energy-producing pathways. These typically include the initial “depletion” of glycogen and glucose stores, followed by a transient hampering of insulin secretion and insulin-dependent glucose uptake (via GLUT4). The associated increased insulin resistance was also demonstrated in the current study by elevated POST 2-hydroxybutyric acid concentrations (Sousa et al., 2021). In turn, this is thought to not only result in intracellular hypoglycemia/extracellular hyperglycemia, but also the activation of alternative fuel substrate catabolism/utilization pathways, such as gluconeogenesis, amino acid catabolism, ketogenesis, and lipolysis (Lewis et al., 2010; Stander et al., 2018; Bester et al., 2021). Moreover, it reported that the significant strain placed on the TCA cycle and electron transport chain further leads to an imbalanced redox potential and the activation of minor energy-producing pathways including α/ω-oxidation and mTOR-mediated substrate release (Stander et al., 2018).

**FIGURE 6:**
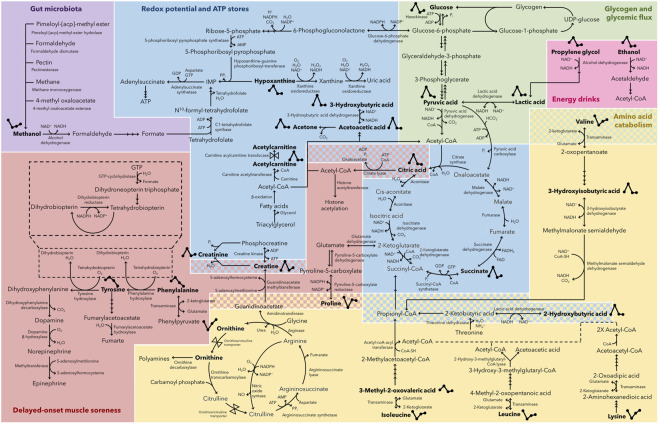
Post-marathon recovering metabolome. Metabolic map indicating the 26 metabolites (in bold) that changed significantly (*p* ≤ 0.05 and *d* ≥ 0.5) during a 48 h post-marathon recovery period. Metabolites are grouped according to their relevance to recovering gut microbiota metabolism (purple), redox potential and ATP store restoration (blue), glycogen resynthesis and glycemic flux restoration (green), the consumption of energy drinks (pink), amino acid metabolism (yellow), and delayed-onset muscle soreness (red). Those metabolites with two-group associations are indicated as dual-colored checkered sections. ATP, adenosine triphosphate; ADP, adenosine diphosphate; AMP, adenosine monophosphate; NAD^+^, nicotinamide adenine dinucleotide; NADH, reduced nicotinamide adenine dinucleotide; NADP^+^, nicotinamide adenine dinucleotide phosphate; NADPH, reduced nicotinamide adenine dinucleotide phosphate; P_i_, inorganic phosphate; CoA, coenzyme A; FAD, flavin adenine dinucleotide; FADH_2_, reduced flavin adenine dinucleotide; GTP, guanosine triphosphate; GDP, guanosine diphosphate; UDP, uracil-diphosphate.

Based on these metabolic adaptations, the current investigation aimed to provide a better understanding of the connatural metabolic recovery trend of athletes over a 48 h period after completing a marathon. This was achieved by 1) confirming whether metabolic recovery to a pre-marathon related state was attained at 24 h and/or 48 h, respectively, and 2) determining the recovery trend of the perturbed metabolome after 24 and 48 h of recovery. The comprehensive significance of these observed metabolic changes (Table 3) is discussed below and schematically represented in Figure 6.

### 4.1 Restoration of redox potential and ATP stores

Upon cessation of endurance exercise, the initial steps of metabolic recovery involves the rapid restoration of myocellular oxygen levels, ATP production, and replenishment of phosphocreatine stores (Egan and Zierath, 2013). In the current investigation, this was confirmed (Table 3; Figure 6) by the reduction in phosphagen catabolism intermediates (creatine and creatinine) and hypoxanthine concentrations to PRE-related levels by D1. Initial POST elevations in hypoxanthine were ascribed to an upregulated purine catabolism during the marathon, as ATP is broken down to adenosine and eventually hypoxanthine. The latter is then further sequentially catabolized to xanthine and uric acid by an oxygen and NAD^+^-dependent multifunctional enzyme collectively referred to as xanthine oxidoreductase (Bortolotti et al., 2021), or may be directly eliminated via urinary excretion (Kelly et al., 2020). As such, a D1 reduction in hypoxanthine concentrations may not only support the aforementioned reduction in ATP catabolism but is also indicative of the amelioration of circulating oxygen levels and intracellular redox potential (Yin et al., 2021). The latter further coincides with a reduction in D1 and D2 concentrations of ketone bodies (3-hydroxybutyric acid, acetoacetic acid, and acetone), 2-hydroxybutyric acid, and TCA-cycle intermediates (citric acid and succinic acid), which were perturbed at POST due to the extensive strain placed on the electron transport chain leading to an imbalanced mitochondrial NAD^+^:NADH ratio during the marathon (Tretter et al., 2016; Koutnik et al., 2019; Rojas-Morales et al., 2020; Sousa et al., 2021). Although NAD^+^ and NADH were not directly measured as part of this investigation, previous literature (Esterhuizen et al., 2017) has suggested that a 3-hydroxybutyric acid:acetoacetic acid ratio may be used as a proportional indicator of the mitochondrial NAD^+^:NADH ratio. In the current investigation, a POST 3-hydroxybutyric acid:acetoacetic acid ratio of 8:1 was observed, which recovered to PRE-related levels of approximately 2:1 by D1. Additionally, the reduction in ketone bodies may be directly associated with a reduction in lipolysis and the subsequent production of acetyl-CoA, as indicated by the reduction in the acetylcarnitine levels to PRE-related concentrations by D1. Finally, carnitine plays a role in the maintenance of the mitochondrial acetyl-CoA/CoA-SH ratio via the carnitine acetyltransferase-catalyzed production of acetylcarnitine during conditions of accumulating acetyl-CoA (Stephens, 2018). Considering this buffering role of carnitine, the rapid normalization of acetylcarnitine further indicates the restoration of the marathon-induced high mitochondrial acetyl-CoA/CoA-SH ratio.

### 4.2 Glycogen resynthesis and glycemic flux restoration

According to previous literature (Egan and Zierath, 2013), muscle glycogen resynthesis is prioritized in the immediate hours following endurance activity and is supported by dietary intake. However, as opposed to the initial rapid restoration of ATP stores, muscle glycogen is only thought to recover to pre-perturbed levels within 24–48 h after cessation of endurance exercise (Figure 6). This coincides with the normalization of glucose and pyruvic acid concentrations (Table 3) to PRE-related concentrations only within 48 h post-marathon (D2) in this investigation. Although not considered statistically significant, glucose initially decreased to below baseline at D1 and normalized at D2, whereas pyruvic acid initially increased at D1 before normalizing at D2. These interim fluctuations may be indicative of a temporary upregulation of post-marathon glycogenesis as a means of restoring glycogen reserves, followed by a rapid stabilization once these reserves have been recovered. The restoration of blood glucose levels is expected to be a result of both post-marathon dietary carbohydrate intake, as well as the subsequent replenishment of liver and muscle glycogen levels. Additionally, rapid convalescence of lactic acid concentrations by D1 (Table 3) highlights the occurrence of gluconeogenesis (via the Cori cycle) and glycogenesis, the former of which provides additional glucose to support the latter and coincides with the initial increased D1 pyruvic acid concentrations (Brooks, 2020).

### 4.3 Amino acid metabolism and delayed-onset muscle soreness

The previously reported upregulation of amino acid catabolism during the marathon (Bester et al., 2021) result in the accumulation of ammonia and consequential upregulated urea cycle (Takeda and Takemasa, 2013). This was confirmed by the reduced POST ornithine concentrations observed in the current study (Table 3) although not significantly so. Furthermore, an endurance exercise-induced upregulated decarboxylation of ornithine (Turchanowa et al., 2000) may result in polyamine accumulation and consequential enhanced muscle recovery (Pitti et al., 2019; Sagar et al., 2021; Schranner et al., 2021). In accordance with previous literature (Stander et al., 2020) and the D1 recovery of ornithine concentrations to PRE-related levels, several amino acids (lysine, proline, isoleucine, and leucine) and associated catabolic intermediates (2-hydroxybutyric acid, 3-hydroxyisobutyric acid, and 3-methyl-2-oxovaleric acid) steadily recovered to PRE-related concentrations by D1, indicating the reduced need to produce energy via amino acid catabolism, increased dietary protein intake, and a metabolic shift towards protein synthesis (Figure 6). Although the recovery trend of valine was similar to that of the aforementioned amino acids, this BCAA did not fully recover by D1 to PRE-related concentrations (*p* = 2.85 × 10^−2^, *d* = 0.54; Supplementary Table S2; Supplementary Figure S3A). The delayed recovery of valine coincides with the aforementioned delay in post-marathon pyruvic acid and glucose recovery, thereby suggesting the involvement of valine in post-marathon glycogen resynthesis (Figure 6). Moreover, valine supplementation has been reported to facilitate post-exercise glycogen resynthesis and improve spontaneous muscle activity (Tsuda et al., 2018). Nevertheless, valine is an essential amino acid, and the involvement of dietary intake should therefore also be considered, not only in the post-marathon recovery of both valine and glycogen, but also in the preservation of muscle glycogen during the endurance event. As opposed to the steady increase in the BCAAs and lysine concentrations between D1 and D2 (although not significantly so), proline concentrations decreased at D2 following its initial recovery at D1 to PRE-related levels (Table 3). Although not considered statistically significant, this reduction may be ascribed to the occurrence of delayed-onset muscle soreness (DOMS; Figure 6). DOMS is typically defined as acute muscle pain experienced between 24 and 72 h following intensive exercise, which peaks after 48 h (Wilke and Behringer, 2021). Accordingly, an elevated serum hydroxyproline concentration was seen by Stander et al. (2020) 48 h post-marathon, indicating the onset of delayed post-marathon collagen breakdown. Additionally, the non-significant D2 elevation in creatine (Table 3) and previously reported elevations in inflammatory markers and epidermal growth factor (Clifford et al., 2017) have respectively been associated with an upregulation in creatine kinase activity and the regulation of muscle anabolism/repair, which further supports the occurrence of DOMS. In addition, the aforementioned reduction in citric acid concentrations within 48 h post-marathon, although not significantly so, may also be attributed to an upregulated ATP-citrate lyase activity. This enzyme has been associated with increased skeletal muscle regeneration and myofiber differentiation via histone acetylation (Das et al., 2017), thereby associating citric acid with DOMS.

In contrast to the previously described amino acids, tyrosine and phenylalanine were elevated after the completion of the marathon. This is ascribed to a reduced liver function (Ishikawa et al., 2017), supported by the elevated post-marathon AST levels previously described by Clifford et al. (2017). Moreover, liver function is directly related to the Fischer’s ratio (Fischer et al., 1976; Chen et al., 2020), which was decreased at POST (1.85) when compared to PRE (2.85) in the current study. Nevertheless, liver function was recovered by D1 (2.63) to PRE-related levels. Additionally, the elevated POST phenylalanine and tyrosine levels may also indicate reduced brain glycogen levels (Matsui et al., 2019), concurring with the marathon-induced initial depletion of glycogen stores. Interestingly, following initial D1 recovery of phenylalanine and tyrosine, their concentrations decreased at D2 (Table 3) relative to PRE levels, with that of tyrosine being statistically significant (*p* = 1.29 × 10^−2^, *d* = 1.20; Supplementary Table S3; Supplementary Figure S4A). This delayed response may also be linked to the aforementioned elevated DOMS-associated inflammatory markers (Lenn et al., 2002). The expression of guanosine-triphosphate-cyclohydrolase-1 is typically upregulated by these inflammatory markers, subsequently leading to the upregulation in tetrahydrobiopterin synthesis (Chen et al., 2011), and consequently the activities of tyrosine hydroxylase and phenylalanine hydroxylase (Capuron et al., 2011). The resultant induced breakdown of tyrosine and phenylalanine may likely increase dopamine, epinephrine, and norepinephrine levels, which have been implicated in the modulation of muscle pain through mechanisms which remain to be elucidated (Brumovsky, 2016; Kirkpatrick et al., 2016; Mizumura and Taguchi, 2016). This suggests the possible involvement of these neurotransmitters, tyrosine, and phenylalanine in DOMS. Of note is the neutralizing effect of ROS on tetrahydrobiopterin and consequential accumulation of tyrosine and phenylalanine, providing an additional explanation for the elevated POST concentrations thereof.

### 4.4 Gut microbiota

Methanol is typically produced endogenously by gut microbiota via anaerobic fermentation (Figure 6, purple) in healthy humans (Dorokhov et al., 2015; Pechlivanis et al., 2015). However, during long-distance aerobic/endurance running, intestinal absorption of this metabolite is reportedly downregulated (Komarova et al., 2014; Walter et al., 2021) due to the diversion of splanchnic blood flow to the working skeletal muscles (de Oliveira et al., 2014). Although not chronic or serious, the marathon-induced gastrointestinal discomfort may result in increased mesenteric ischemia and decreased gastrointestinal perfusion, thus ascribing the initial reduction of POST serum methanol concentrations. Moreover, the severity of these endurance running-induced deleterious gastrointestinal symptoms is associated with the distance and intensity of the endurance race (Pfeiffer et al., 2012), and the associated impaired gut function affects runners even during the post-marathon recovery stage (de Oliveira et al., 2014). Accordingly, methanol concentrations gradually increased at D1 and D2 in the current study (Table 3) but did not recover to PRE-related levels within 24 h after the marathon (*p* = 5.13 × 10^−3^, *d* = 2.67; Supplementary Table S2; Supplementary Figure S3B). Furthermore, various environmental factors have a large effect on the severity of the gastrointestinal changes occurring during a marathon, and higher temperatures have been related to an increased prevalence of dehydration and gastrointestinal damage (Simons and Kennedy, 2004; Walter et al., 2021). However, considering the relatively cold temperatures during the course of the marathon in this study (Table 2), extensive gastrointestinal damage would have been unlikely, supported by the rapid recovery of methanol by D2 (Table 3).

### 4.5 Consumption of energy drinks

Initial elevations in ethanol and propylene glycol directly after the marathon (POST) may be ascribed to 1) the consumption of energy drinks during the marathon, as many ingredients thereof (such as ginseng) are dissolved in small amounts of these alcohols (Lutmer et al., 2009); and 2) ineffective clearance via alcohol dehydrogenase during endurance exercise (Choi and So, 2018). Although ethanol and propylene glycol concentrations decreased significantly within 24 h post-marathon, only propylene glycol recovered to PRE-related levels (Table 3). Ethanol reductions were more prominent and remained well below PRE levels at both D1 (*p* = 3.68 × 10^−4^, *d* = 2.67; Supplementary Table S2; Supplementary Figure S3C) and D2 (*p* = 2.73 × 10^−4^, *d* = 2.22; Supplementary Table S3; Supplementary Figure S4B). This may be ascribed to elevated post-marathon ethanol elimination due to vasodilation and hydration. Additionally, post-marathon ethanol breakdown may aid in acetyl-CoA production and the recovery of NAD:NADH ratios, ATP, and fuel substrate stores (Figure 6).

## 5 Limitations and future perspectives

Inter-individual variability is an unavoidable limitation in any study involving human participants. As such, the current study employed a paired univariate statistical design to select significant features, thereby ensuring that each participant served as their own control. Additionally, adding dietary restrictions to the already refrained use of recovery aids was considered too much of an interference to the personalized regimens of the study participants. Nevertheless, dietary influences were considered as possible influencing factors during interpretation of the identified metabolite changes. Considering that alanine and glycine were not detected in the current investigation, further targeted investigation into the post-marathon recovering amino acid profile should be done to affirm whether alanine and glycine, which are common metabolites in serum, play a significant role in recovery. Furthermore, lipid profiles were not detected in the current investigation, likely as a result of the polar buffer solution that contained a polar locking compound, namely, deuterium oxide. Future, more targeted examinations of the non-polar metabolite classes (e.g., lipids) using ^1^H-NMR metabolomics would significantly aid in providing a more comprehensive view of the post-marathon recovering metabolome. However, this would require additional sample preparation steps to extract these non-polar metabolites, thereby incorporating additional analytical steps that may inevitably invite cumulative analytical variation and negate the proficient analytical repeatability associated with ^1^H-NMR. Finally, energy drink consumption was not monitored during the marathon run of the current investigation. As such, the metabolic implications of energy drink consumption during these exhaustive exercises may also hold promising insights and should be further evaluated.

Future studies may consider conducting in-depth statistical correlations between the metabolic fluctuations associated with post-marathon recovery and exercise performance parameters to further aid in the development of personalized recovery strategies. Moreover, the identification of those metabolites associated with recovering neurotransmitter and aromatic amino acid levels, collagen breakdown, muscle damage, and histone acetylation may be a promising direction for future metabolomics studies. While doing so, it would be beneficial to extend the monitored recovery period to at least 72 h following aerobic/endurance exercise, thereby providing a better understanding of the complete recovery process of the post-marathon metabolome, and determine optimal time points for sample collection for similar studies in the future. Additionally, while methanol has largely been regarded as a metabolic waste molecule or contaminant, statistically significant normalization of this metabolite was observed in this investigation, suggesting that it may play a role in normal metabolic processes and warrants further investigation. Nevertheless, low concentrations of this metabolite are present in healthy human serum, the rapid normalization of which, as seen herein, suggests that it does play a role in normal metabolic processes. An additional consideration is that the urinary elimination of certain metabolites (e.g., hypoxanthine, lactate, and pyruvate) could be contributing to the post-marathon recovering metabolome. Therefore, using both serum and urine collected from the same cohort, at the same times, could provide valuable information regarding which metabolites are recovering due to dietary intake, inherent recovery mechanisms, or increased urinary excretion.

## 6 Conclusion

In the current investigation, a recovered post-marathon serum metabolome may be defined as the restoration of the perturbation-induced altered metabolic flux and associated serum metabolite concentrations to their pre-perturbation/pre-marathon levels. In the case of exercise, this involves the recovery of metabolic fuel substrate levels, ATP stores, and the amelioration of other exercise induced physiological changes including, but not limited to, gastrointestinal, muscle, liver, kidney, and cardiovascular damage. To summarize, the current study utilized an untargeted ^1^H-NMR metabolomics approach to evaluate the recovering serum metabolome following a marathon by characterizing those metabolites (26 distinct metabolites in total) that 1) did not recover (*n* = 4) and 2) changed significantly (*n* = 25) within 24 and 48 h post-marathon. This allowed for the identification of several metabolites (proline, creatine, citric acid, phenylalanine, and tyrosine) possibly associated with DOMS, which is commonly experienced by marathon athletes irrespective of their training status. Finally, the metabolite markers identified in this study and the metabolic recovery mechanisms described by these gives clues to improved recovery strategies, which may include pre- and post-training/race supplementation with phenylalanine, tyrosine, valine, and/or proline. Furthermore, these “late-stage recovery” metabolite markers may also be used for personalized monitoring of athletic health/recovery/performance.

## Data Availability

The raw data supporting the conclusions of this article will be made available by the corresponding author upon reasonable request, without undue reservation.

## References

[B1] Ambrozic G. Udovc G. Krusic P. (2018). IAAF competition rules 2018-2019. Ljubljana: Athletic Association of Slovenia.

[B2] Barros E. S. Nascimento D. C. Prestes J. Nobrega O. T. Cordova C. Sousa F. (2017). Acute and chronic effects of endurance running on inflammatory markers: A systematic review. Front. Physiol. 8, 779. 10.3389/fphys.2017.00779 29089897 PMC5650970

[B3] Bernat-Adell M. D. Collado-Boira E. J. Moles-Julio P. Panizo-Gonzalez N. Martinez-Navarro I. Hernando-Fuster B. (2021). Recovery of inflammation, cardiac, and muscle damage biomarkers after running a marathon. J. Strength Cond. Res. 35 (3), 626–632. 10.1519/JSC.0000000000003167 31045685

[B4] Bester R. Stander Z. Mason S. Keane K. M. Howatson G. Clifford T. (2021). Characterizing marathon-induced metabolic changes using (1)H-NMR metabolomics. Metabolites 11 (10), 656. 10.3390/metabo11100656 34677371 PMC8541139

[B5] Bortolotti M. Polito L. Battelli M. G. Bolognesi A. (2021). Xanthine oxidoreductase: One enzyme for multiple physiological tasks. Redox Biol. 41, 101882. 10.1016/j.redox.2021.101882 33578127 PMC7879036

[B6] Brooks G. A. (2020). Lactate as a fulcrum of metabolism. Redox Biol. 35, 101454. 10.1016/j.redox.2020.101454 32113910 PMC7284908

[B7] Brumovsky P. R. (2016). Dorsal root ganglion neurons and tyrosine hydroxylase--an intriguing association with implications for sensation and pain. Pain 157 (2), 314–320. 10.1097/j.pain.0000000000000381 26447702 PMC4727984

[B8] Capuron L. Schroecksnadel S. Feart C. Aubert A. Higueret D. Barberger-Gateau P. (2011). Chronic low-grade inflammation in elderly persons is associated with altered tryptophan and tyrosine metabolism: Role in neuropsychiatric symptoms. Biol. Psychiatry 70 (2), 175–182. 10.1016/j.biopsych.2010.12.006 21277567

[B9] Chen S. H. Wan Q. S. Wang T. Zhang K. H. (2020). Fluid biomarkers for predicting the prognosis of liver cirrhosis. Biomed. Res. Int. 2020, 7170457. 10.1155/2020/7170457 32280697 PMC7114768

[B10] Chen W. Li L. Brod T. Saeed O. Thabet S. Jansen T. (2011). Role of increased guanosine triphosphate cyclohydrolase-1 expression and tetrahydrobiopterin levels upon T cell activation. J. Biol. Chem. 286 (16), 13846–13851. 10.1074/jbc.M110.191023 21343293 PMC3077585

[B11] Choi E. J. So W. Y. (2018). Effects of exercise intensity on alcohol dehydrogenase gene expression in the rat large IntestineExercise and ADH gene expression. J. Men's Health 14 (2). 10.22374/1875-6859.14.2.2

[B12] Clifford T. Allerton D. M. Brown M. A. Harper L. Horsburgh S. Keane K. M. (2017). Minimal muscle damage after a marathon and no influence of beetroot juice on inflammation and recovery. Appl. Physiol. Nutr. Metab. 42 (3), 263–270. 10.1139/apnm-2016-0525 28165768

[B13] D'Silva A. Bhuva A. N. van Zalen J. Bastiaenen R. Abdel-Gadir A. Jones S. (2020). Cardiovascular remodeling experienced by real-world, unsupervised, young novice marathon runners. Front. Physiol. 11, 232. 10.3389/fphys.2020.00232 32256389 PMC7093496

[B14] Dalle Carbonare L. Mottes M. Cheri S. Deiana M. Zamboni F. Gabbiani D. (2019). Increased gene expression of RUNX2 and SOX9 in mesenchymal circulating progenitors is associated with autophagy during physical activity. Oxid. Med. Cell. Longev. 2019, 8426259. 10.1155/2019/8426259 31737174 PMC6815530

[B15] Das S. Morvan F. Morozzi G. Jourde B. Minetti G. C. Kahle P. (2017). ATP citrate lyase regulates myofiber differentiation and increases regeneration by altering histone acetylation. Cell. Rep. 21 (11), 3003–3011. 10.1016/j.celrep.2017.11.038 29241530

[B16] de Oliveira E. P. Burini R. C. Jeukendrup A. (2014). Gastrointestinal complaints during exercise: Prevalence, etiology, and nutritional recommendations. Sports Med. 44 (1), S79–S85. 10.1007/s40279-014-0153-2 24791919 PMC4008808

[B17] Dorokhov Y. L. Shindyapina A. V. Sheshukova E. V. Komarova T. V. (2015). Metabolic methanol: Molecular pathways and physiological roles. Physiol. Rev. 95 (2), 603–644. 10.1152/physrev.00034.2014 25834233

[B18] Dunn W. B. Ellis D. I. (2005). Metabolomics: Current analytical platforms and methodologies. TrAC Trends Anal. Chem. 24 (4), 285–294. 10.1016/j.trac.2004.11.021

[B19] Egan B. Zierath J. R. (2013). Exercise metabolism and the molecular regulation of skeletal muscle adaptation. Cell. Metab. 17 (2), 162–184. 10.1016/j.cmet.2012.12.012 23395166

[B20] Eigenvector Research (2021). PLS_Toolbox". 9.0. Manson, Washington: Eigenvector Research, Inc.

[B21] Esterhuizen K. van der Westhuizen F. H. Louw R. (2017). Metabolomics of mitochondrial disease. Mitochondrion 35, 97–110. 10.1016/j.mito.2017.05.012 28576558

[B22] Fischer J. E. Rosen H. M. Ebeid A. M. James J. H. Keane J. M. Soeters P. B. (1976). The effect of normalization of plasma amino acids on hepatic encephalopathy in man. Surgery 80 (1), 77–91. 818729

[B23] Hammer C. Podlog L. (2016). “Motivation and marathon running,” in Marathon running: Physiology, psychology, nutrition and training aspects (Germany: Springer International Publishing), 107–124.

[B24] Hargreaves M. Spriet L. L. (2020). Skeletal muscle energy metabolism during exercise. Nat. Metab. 2 (9), 817–828. 10.1038/s42255-020-0251-4 32747792

[B25] Hawley J. A. Leckey J. J. (2015). Carbohydrate dependence during prolonged, intense endurance exercise. Sports Med. 45 (1), S5–S12. 10.1007/s40279-015-0400-1 26553495 PMC4672006

[B26] Ialongo C. (2016). Understanding the effect size and its measures. Biochem. Med. Zagreb. 26 (2), 150–163. 10.11613/BM.2016.015 27346958 PMC4910276

[B27] Ishikawa T. Imai M. Ko M. Sato H. Nozawa Y. Sano T. (2017). Evaluation of the branched-chain amino acid-to-tyrosine ratio prior to treatment as a prognostic predictor in patients with liver cirrhosis. Oncotarget 8 (45), 79480–79490. 10.18632/oncotarget.18447 29108327 PMC5668060

[B28] Kellmann M. Bertollo M. Bosquet L. Brink M. Coutts A. J. Duffield R. (2018). Recovery and performance in Sport: Consensus statement. Int. J. Sports Physiol. Perform. 13 (2), 240–245. 10.1123/ijspp.2017-0759 29345524

[B29] Kelly R. S. Kelly M. P. Kelly P. (2020). Metabolomics, physical activity, exercise and health: A review of the current evidence. Biochim. Biophys. Acta Mol. Basis Dis. 1866 (12), 165936. 10.1016/j.bbadis.2020.165936 32827647 PMC7680392

[B30] Kirkpatrick D. R. McEntire D. M. Smith T. A. Dueck N. P. Kerfeld M. J. Hambsch Z. J. (2016). Transmission pathways and mediators as the basis for clinical pharmacology of pain. Expert Rev. Clin. Pharmacol. 9 (10), 1363–1387. 10.1080/17512433.2016.1204231 27322358 PMC5215101

[B31] Komarova T. V. Petrunia I. V. Shindyapina A. V. Silachev D. N. Sheshukova E. V. Kiryanov G. I. (2014). Endogenous methanol regulates mammalian gene activity. PLoS One 9 (2), e90239. 10.1371/journal.pone.0090239 24587296 PMC3937363

[B32] Koutnik A. P. D'Agostino D. P. Egan B. (2019). Anticatabolic effects of ketone bodies in skeletal muscle. Trends Endocrinol. Metab. 30 (4), 227–229. 10.1016/j.tem.2019.01.006 30712977

[B33] Kwiecien S. Y. McHugh M. P. Hicks K. M. Keane K. M. Howatson G. (2021). Prolonging the duration of cooling does not enhance recovery following a marathon. Scand. J. Med. Sci. Sports 31 (1), 21–29. 10.1111/sms.13822 32901996

[B34] Lenn J. Uhl T. Mattacola C. Boissonneault G. Yates J. Ibrahim W. (2002). The effects of fish oil and isoflavones on delayed onset muscle soreness. Med. Sci. Sports Exerc. 34 (10), 1605–1613. 10.1097/00005768-200210000-00012 12370562

[B35] Lewis G. D. Farrell L. Wood M. J. Martinovic M. Arany Z. Rowe G. C. (2010). Metabolic signatures of exercise in human plasma. Sci. Transl. Med. 2 (33), 33ra37. 10.1126/scitranslmed.3001006 PMC3010398 20505214

[B36] Lutmer B. Zurfluh C. Long C. (2009). Potential effect of alcohol content in energy drinks on breath alcohol testing. J. Anal. Toxicol. 33 (3), 167–169. 10.1093/jat/33.3.167 19371466

[B37] Manier S. K. Meyer M. R. (2020). Current situation of the metabolomics techniques used for the metabolism studies of new psychoactive substances. Ther. Drug Monit. 42 (1), 93–97. 10.1097/FTD.0000000000000694 31425443

[B38] Martinez-Navarro I. Montoya-Vieco A. Hernando C. Hernando B. Panizo N. Collado E. (2021). The week after running a marathon: Effects of running vs elliptical training vs resting on neuromuscular performance and muscle damage recovery. Eur. J. Sport Sci. 21 (12), 1668–1674. 10.1080/17461391.2020.1857441 33251988

[B39] Mason S. Terburgh K. Louw R. (2018). Miniaturized (1)H-NMR method for analyzing limited-quantity samples applied to a mouse model of Leigh disease. Metabolomics 14 (6), 74. 10.1007/s11306-018-1372-6 30830372

[B40] Matsui T. Liu Y. F. Soya M. Shima T. Soya H. (2019). Tyrosine as a mechanistic-based biomarker for brain glycogen decrease and supercompensation with endurance exercise in rats: A metabolomics study of plasma. Front. Neurosci. 13, 200. 10.3389/fnins.2019.00200 30941004 PMC6433992

[B41] Mizumura K. Taguchi T. (2016). Delayed onset muscle soreness: Involvement of neurotrophic factors. J. Physiol. Sci. 66 (1), 43–52. 10.1007/s12576-015-0397-0 26467448 PMC10716961

[B42] Moler C. (2021). MATLAB". 9.11.0.1837725. Natrick, Massachusetts: The MathWorks Inc.

[B43] Moore D. R. (2019). Maximizing post-exercise anabolism: The case for relative protein intakes. Front. Nutr. 6, 147. 10.3389/fnut.2019.00147 31552263 PMC6746967

[B44] Nieman D. C. (2007). Marathon training and immune function. Sports Med. 37 (4-5), 412–415. 10.2165/00007256-200737040-00036 17465622

[B45] Nieman D. C. Mitmesser S. H. (2017). Potential impact of nutrition on immune system recovery from heavy exertion: A metabolomics perspective. Nutrients 9 (5), 513. 10.3390/nu9050513 28524103 PMC5452243

[B46] Nieman D. C. Shanely R. A. Gillitt N. D. Pappan K. L. Lila M. A. (2013). Serum metabolic signatures induced by a three-day intensified exercise period persist after 14 h of recovery in runners. J. Proteome Res. 12 (10), 4577–4584. 10.1021/pr400717j 23984841

[B47] Pang Z. Chong J. Zhou G. de Lima Morais D. A. Chang L. Barrette M. (2021). MetaboAnalyst 5.0: Narrowing the gap between raw spectra and functional insights. Nucleic Acids Res. 49 (W1), W388–W396. 10.1093/nar/gkab382 34019663 PMC8265181

[B48] Pechlivanis A. Papaioannou K. G. Tsalis G. Saraslanidis P. Mougios V. Theodoridis G. A. (2015). Monitoring the response of the human urinary metabolome to brief maximal exercise by a combination of RP-UPLC-MS and (1)H NMR spectroscopy. J. Proteome Res. 14 (11), 4610–4622. 10.1021/acs.jproteome.5b00470 26419189

[B49] Pfeiffer B. Stellingwerff T. Hodgson A. B. Randell R. Pottgen K. Res P. (2012). Nutritional intake and gastrointestinal problems during competitive endurance events. Med. Sci. Sports Exerc 44 (2), 344–351. 10.1249/MSS.0b013e31822dc809 21775906

[B50] Philippou A. Chryssanthopoulos C. Maridaki M. Dimitriadis G. Koutsilieris M. (2019). “Exercise metabolism in health and disease,” in Cardiorespiratory fitness in cardiometabolic diseases (Germany: Springer International Publishing), 57–96.

[B51] Pitti E. Petrella G. Di Marino S. Summa V. Perrone M. D'Ottavio S. (2019). Salivary metabolome and soccer match: Challenges for understanding exercise induced changes. Metabolites 9 (7), 141. 10.3390/metabo9070141 31336760 PMC6680540

[B52] Rojas-Morales P. Pedraza-Chaverri J. Tapia E. (2020). Ketone bodies, stress response, and redox homeostasis. Redox Biol. 29, 101395. 10.1016/j.redox.2019.101395 31926621 PMC6911969

[B53] Sagar N. A. Tarafdar S. Agarwal S. Tarafdar A. Sharma S. (2021). Polyamines: Functions, metabolism, and role in human disease management. Med. Sci. (Basel) 9 (2), 44. 10.3390/medsci9020044 34207607 PMC8293435

[B54] Sakaguchi C. A. Nieman D. C. Signini E. F. Abreu R. M. Catai A. M. (2019). Metabolomics-based studies assessing exercise-induced alterations of the human metabolome: A systematic review. Metabolites 9 (8), 164. 10.3390/metabo9080164 31405020 PMC6724094

[B55] Saunders M. J. Luden N. D. DeWitt C. R. Gross M. C. Dillon Rios A. (2018). Protein supplementation during or following a marathon run influences post-exercise recovery. Nutrients 10 (3), 333. 10.3390/nu10030333 29534444 PMC5872751

[B56] Schader J. F. Haid M. Cecil A. Schoenfeld J. Halle M. Pfeufer A. (2020). Metabolite shifts induced by marathon race competition differ between athletes based on level of fitness and performance: A substudy of the enzy-MagIC study. Metabolites 10 (3), 87. 10.3390/metabo10030087 32121570 PMC7143325

[B57] Schranner D. Schonfelder M. Romisch-Margl W. Scherr J. Schlegel J. Zelger O. (2021). Physiological extremes of the human blood metabolome: A metabolomics analysis of highly glycolytic, oxidative, and anabolic athletes. Physiol. Rep. 9 (12), e14885. 10.14814/phy2.14885 34152092 PMC8215680

[B58] Schrimpe-Rutledge A. C. Codreanu S. G. Sherrod S. D. McLean J. A. (2016). Untargeted metabolomics strategies-challenges and emerging directions. J. Am. Soc. Mass Spectrom. 27 (12), 1897–1905. 10.1007/s13361-016-1469-y 27624161 PMC5110944

[B59] Simons S. M. Kennedy R. G. (2004). Gastrointestinal problems in runners. Curr. Sports Med. Rep. 3 (2), 112–116. 10.1249/00149619-200404000-00011 14980141

[B60] Smilde A. K. Jansen J. J. Hoefsloot H. C. Lamers R. J. van der Greef J. Timmerman M. E. (2005). ANOVA-Simultaneous component analysis (ASCA): A new tool for analyzing designed metabolomics data. Bioinformatics 21 (13), 3043–3048. 10.1093/bioinformatics/bti476 15890747

[B61] Sousa A. P. Cunha D. M. Franco C. Teixeira C. Gojon F. Baylina P. (2021). Which role plays 2-hydroxybutyric acid on insulin resistance? Metabolites 11 (12), 835. 10.3390/metabo11120835 34940595 PMC8703345

[B62] Sperlich B. (2016). “Physiological aspects of marathon running,” in Marathon running: Physiology, psychology, nutrition and training aspects. Editors Zinner C. Sperlich B. (Switzerland: Springer), 1–12.

[B63] Stander Z. Luies L. Mienie L. J. Keane K. M. Howatson G. Clifford T. (2018). The altered human serum metabolome induced by a marathon. Metabolomics 14 (11), 150. 10.1007/s11306-018-1447-4 30830390

[B64] Stander Z. Luies L. Mienie L. J. Van Reenen M. Howatson G. Keane K. M. (2020). The unaided recovery of marathon-induced serum metabolome alterations. Sci. Rep. 10 (1), 11060. 10.1038/s41598-020-67884-9 32632105 PMC7338546

[B65] Stephens F. B. (2018). Does skeletal muscle carnitine availability influence fuel selection during exercise? Proc. Nutr. Soc. 77 (1), 11–19. 10.1017/S0029665117003937 29037265

[B66] Stöggl T. Wunsch T. (2016). “Biomechanics of marathon running,” in Marathon running: Physiology, psychology, nutrition and training aspects. Editors Zinner C. Sperlich B. (Switzerland: Springer), 13–45.

[B67] Takeda K. Takemasa T. (2013). “An overview of ornithine, arginine and citrulline in exercise and sports nutrition,” in Nutrition and enhanced sports performance (Netherlands: Elsevier Science), 423–431.

[B68] Tretter L. Patocs A. Chinopoulos C. (2016). Succinate, an intermediate in metabolism, signal transduction, ROS, hypoxia, and tumorigenesis. Biochim. Biophys. Acta 1857 (8), 1086–1101. 10.1016/j.bbabio.2016.03.012 26971832

[B69] Tsuda Y. Iwasawa K. Yamaguchi M. (2018). Acute supplementation of valine reduces fatigue during swimming exercise in rats. Biosci. Biotechnol. Biochem. 82 (5), 856–861. 10.1080/09168451.2018.1438168 29475409

[B70] Turchanowa L. Rogozkin V. A. Milovic V. Feldkoren B. I. Caspary W. F. Stein J. (2000). Influence of physical exercise on polyamine synthesis in the rat skeletal muscle. Eur. J. Clin. Invest. 30 (1), 72–78. 10.1046/j.1365-2362.2000.00586.x 10620005

[B71] Walter E. Gibson O. R. Stacey M. Hill N. Parsons I. T. Woods D. (2021). Changes in gastrointestinal cell integrity after marathon running and exercise-associated collapse. Eur. J. Appl. Physiol. 121 (4), 1179–1187. 10.1007/s00421-021-04603-w 33512586

[B72] Wilke J. Behringer M. (2021). Is "delayed onset muscle soreness" a false friend? The potential implication of the fascial connective tissue in post-exercise discomfort. Int. J. Mol. Sci. 22 (17), 9482. 10.3390/ijms22179482 34502387 PMC8431437

[B73] Wilson L. J. Cockburn E. Paice K. Sinclair S. Faki T. Hills F. A. (2018). Recovery following a marathon: A comparison of cold water immersion, whole body cryotherapy and a placebo control. Eur. J. Appl. Physiol. 118 (1), 153–163. 10.1007/s00421-017-3757-z 29127510

[B74] Yin C. Ma Z. Li F. Duan C. Yuan Y. Zhu C. (2021). Hypoxanthine induces muscular ATP depletion and fatigue via UCP2. Front. Physiol. 12, 647743. 10.3389/fphys.2021.647743 33746782 PMC7966526

[B75] Zwanenburg G. Hoefsloot H. C. J. Westerhuis J. A. Jansen J. J. Smilde A. K. (2011). ANOVA-Principal component analysis and ANOVA-simultaneous component analysis: A comparison. J. Chemom. 25 (10), 561–567. 10.1002/cem.1400

