# Supplementary material for: The metabolic recovery of marathon runners: an untargeted 1H-NMR metabolomics perspective
Source: Front Physiol. 2023 May 4;14:1117687. doi: 10.3389/fphys.2023.1117687 (PMC10192615; doi:10.3389/fphys.2023.1117687)
Supplement: Supplementary file 1 [file DataSheet1.pdf]

Supplementary Material

Supplementary Table 1: ^{1}H-NMR metabolite assignments

Metabolite	Center peak chemical shift (ppm)	Protons (n)	Multiplicity	Chemical moiety
2-hydroxybutyric acid	0.90	3	t	CH_3
3-hydroxybutyric acid	1.21	3	d	CH_3
3-hydroxyisobutyric acid	1.07	3	d	CH_3
3-methyl-2-oxovaleric acid	1.10	3	d	CH_3
Acetoacetic acid	2.28	3	s	CH_3
Acetone	2.23	6	s	$(CH_3)_2$
Acetylcarnitine	3.20	9	s	$(CH_3)_3$
Citric acid	2.53	2	d	CH_2
Creatine	3.93	2	s	CH_2
Creatinine	4.06	2	s	CH_2
Ethanol	1.18	3	t	CH_3
α-glucose	5.24	1	d	CH
ß-glucose	4.65	1	d	CH
Hypoxanthine	8.21	2	s	$(CH)_2$
Isoleucine	1.01	3	d	CH_3
Lactic acid	1.33	3	d	CH_3
Leucine	0.97	6	t	$(CH_3)_2$
Lysine	3.02	2	t	CH_2
Methanol	3.37	3	s	CH_3
Ornithine	3.07	2	t	CH_2
Phenylalanine	7.39	5	m	$(CH)_5$
Proline	2.01	2	m	CH_2
Propylene glycol	1.14	3	d	CH_3
Pyruvic acid	2.38	3	s	CH_3
Succinic acid	2.40	4	s	$(CH_2)_2$
Tyrosine	6.91	2	m	$(CH)_2$
Valine	1.04	3	d	CH_3

Supplementary Table 2: Early-stage recovery confirmation. Univariate results indicating statistically significant metabolites which did not recover within 24 h after the marathon (D1), as compared to baseline measurements (PRE).

Metabolite (PubChem ID)	PRE vs D1		
	**Increase/decrease concentration**	***p*-value (< 0.05)**	***d*-value (≥ 0.5)**
Ethanol (702)	Decrease	3.68×10^{-4}	2.67
Methanol (887)	Decrease	5.13×10^{-3}	2.67
Valine (6287)	Decrease	2.85×10^{-2}	0.54

Supplementary Table 3: Late-stage recovery confirmation. Univariate results indicating statistically significant metabolites which did not recover within 48 h after the marathon (D2), as compared to baseline measurements (PRE).

Metabolite (PubChem ID)	PRE vs D2		
	**Increased/decreased concentration**	***p*-value (< 0.05)**	***d*-value (≥ 0.5)**
Ethanol (702)	Decrease	2.73×10^{-4}	2.22
Tyrosine (6057)	Decrease	1.29×10^{-2}	1.20

Supplementary Table 4: Early-stage recovery trend. Univariate results indicating metabolites which changed significantly within the first 24 h after the marathon (D1), as compared to metabolite concentrations directly after the marathon (POST).

Metabolite (PubChem ID)	POST vs D1		
	**Increased/decreased concentration**	***p*-value (< 0.05)**	***d*-value (≥ 0.5)**
2-hydroxybutyric acid (11266)	Decrease	5.69×10^{-5}	2.19
3-hydroxybutyric acid (441)	Decrease	5.69×10^{-5}	2.67
3-hydroxyisobutyric acid (87)	Decrease	1.68×10^{-5}	2.56
3-methyl-2-oxovaleric acid (47)	Decrease	4.46×10^{-2}	0.75
Acetoacetic acid (96)	Decrease	6.28×10^{-5}	1.59
Acetone (180)	Decrease	8.44×10^{-3}	0.87
Acetylcarnitine (7045767)	Decrease	5.26×10^{-7}	3.24
Citric acid (311)	Decrease	2.64×10^{-6}	1.64
Creatine (586)	Decrease	4.03×10^{-4}	1.13

Creatinine (588)	Decrease	1.36×10^{-3}	1.00
Ethanol (702)	Decrease	2.47×10^{-4}	1.84
Glucose (5793)	Decrease	6.45×10^{-3}	1.21
Hypoxanthine (135398638)	Decrease	6.35×10^{-7}	2.67
Isoleucine (6306)	Increase	5.78×10^{-3}	0.78
Lactic acid (612)	Decrease	6.28×10^{-5}	1.98
Leucine (6106)	Increase	1.09×10^{-2}	0.59
Lysine (5962)	Increase	5.78×10^{-3}	1.10
Proline (145742)	Increase	1.04×10^{-3}	1.07
Propylene glycol (1030)	Decrease	1.68×10^{-2}	1.02
Pyruvic acid (1060)	Decrease	3.50×10^{-4}	1.42
Succinic acid (1110)	Decrease	1.22×10^{-4}	1.48
Valine (6287)	Increase	7.28×10^{-3}	0.64

Supplementary Table 5: Late-stage recovery trend. Univariate results indicating metabolites which changed significantly within 48 h after the marathon (D2), as compared to metabolite concentrations directly after the marathon (POST).

**Metabolite (PubChem ID)**	**POST vs D2**		
	**Increased/decreased concentration**	***p*-value (< 0.05)**	***d*-value (≥ 0.5)**
2-hydroxybutyric acid (11266)	Decrease	3.98×10^{-5}	2.21
3-hydroxybutyric acid (441)	Decrease	1.36×10^{-6}	2.60
3-hydroxyisobutyric acid (87)	Decrease	2.90×10^{-5}	2.51
Acetoacetic acid (96)	Decrease	6.16×10^{-5}	1.58
Acetone (180)	Decrease	6.39×10^{-3}	0.94
Acetylcarnitine (7045767)	Decrease	5.87×10^{-6}	3.21
Citric acid (311)	Decrease	8.09×10^{-6}	1.85
Creatine (586)	Decrease	4.45×10^{-2}	0.79
Creatinine (588)	Decrease	6.39×10^{-3}	0.75
Ethanol (702)	Decrease	1.76×10^{-4}	1.63
Isoleucine (6306)	Increase	5.50×10^{-3}	0.85
Lactic acid (612)	Decrease	5.66×10^{-4}	1.95
Leucine (6106)	Increase	9.10×10^{-3}	0.72
Lysine (5962)	Increase	5.53×10^{-4}	1.61

Ornithine (389)	Increase	6.57×10^{-3}	1.00
Phenylalanine (6140)	Decrease	1.06×10^{-2}	0.65
Proline (145742)	Increase	1.46×10^{-3}	0.94
Propylene glycol (1030)	Decrease	3.58×10^{-2}	1.07
Pyruvic acid (1060)	Decrease	1.17×10^{-3}	1.78
Succinic acid (1110)	Decrease	9.25×10^{-4}	1.18
Tyrosine (6057)	Decrease	6.14×10^{-3}	1.18
Valine (6287)	Increase	2.98×10^{-3}	0.86

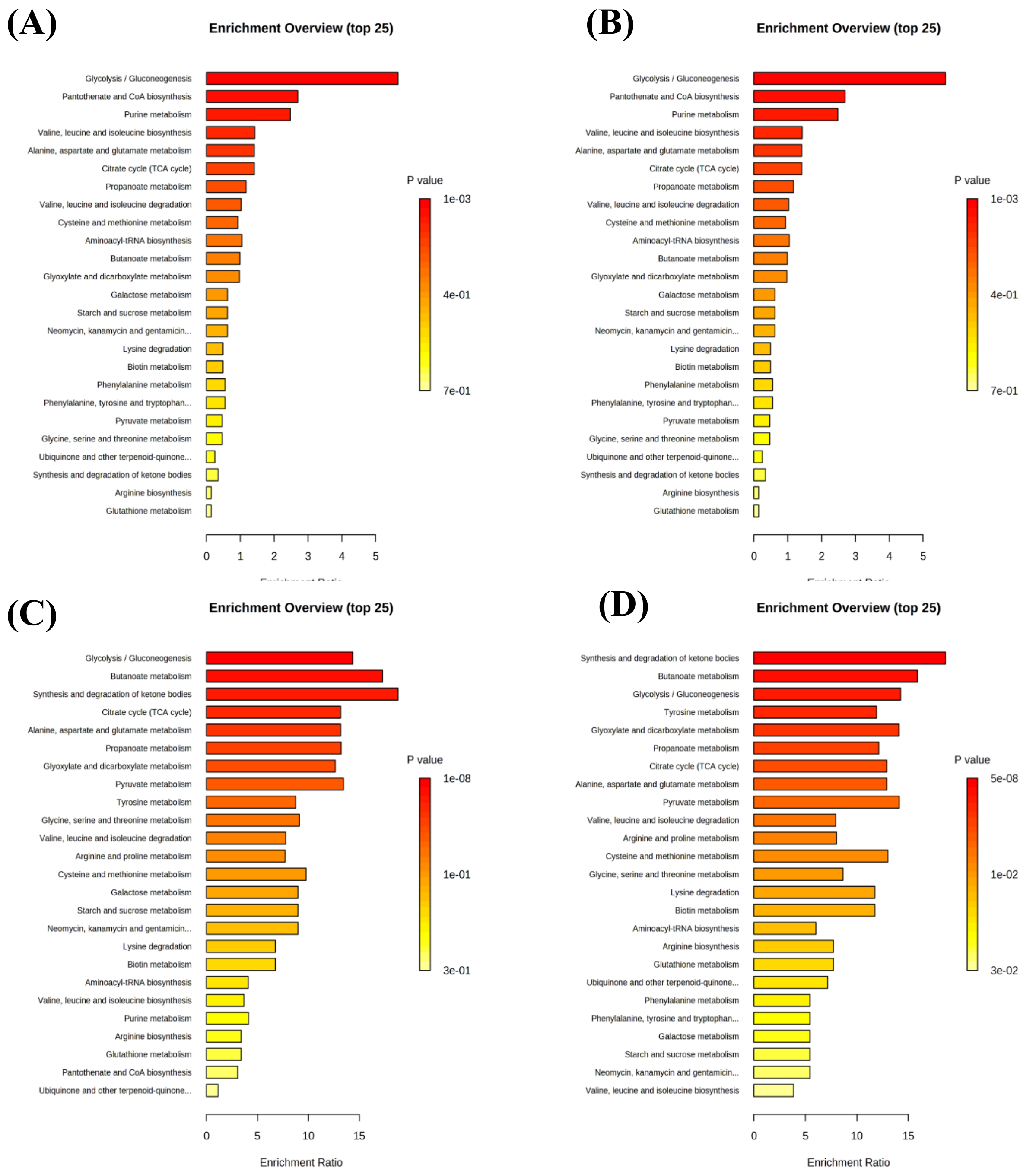

Figure S1: Quantitative enrichment analyses results showing top 25 metabolic pathways identified using the Kyoto Encyclopedia of Genes and Genomes database as reference. Pre-marathon profiles were compared to **(A)** 24 h and **(B)** 48 h post-marathon profiles, and immediately post-marathon profiles were compared to **(C)** and 24 h and **(D)** 48 h post-marathon profiles.

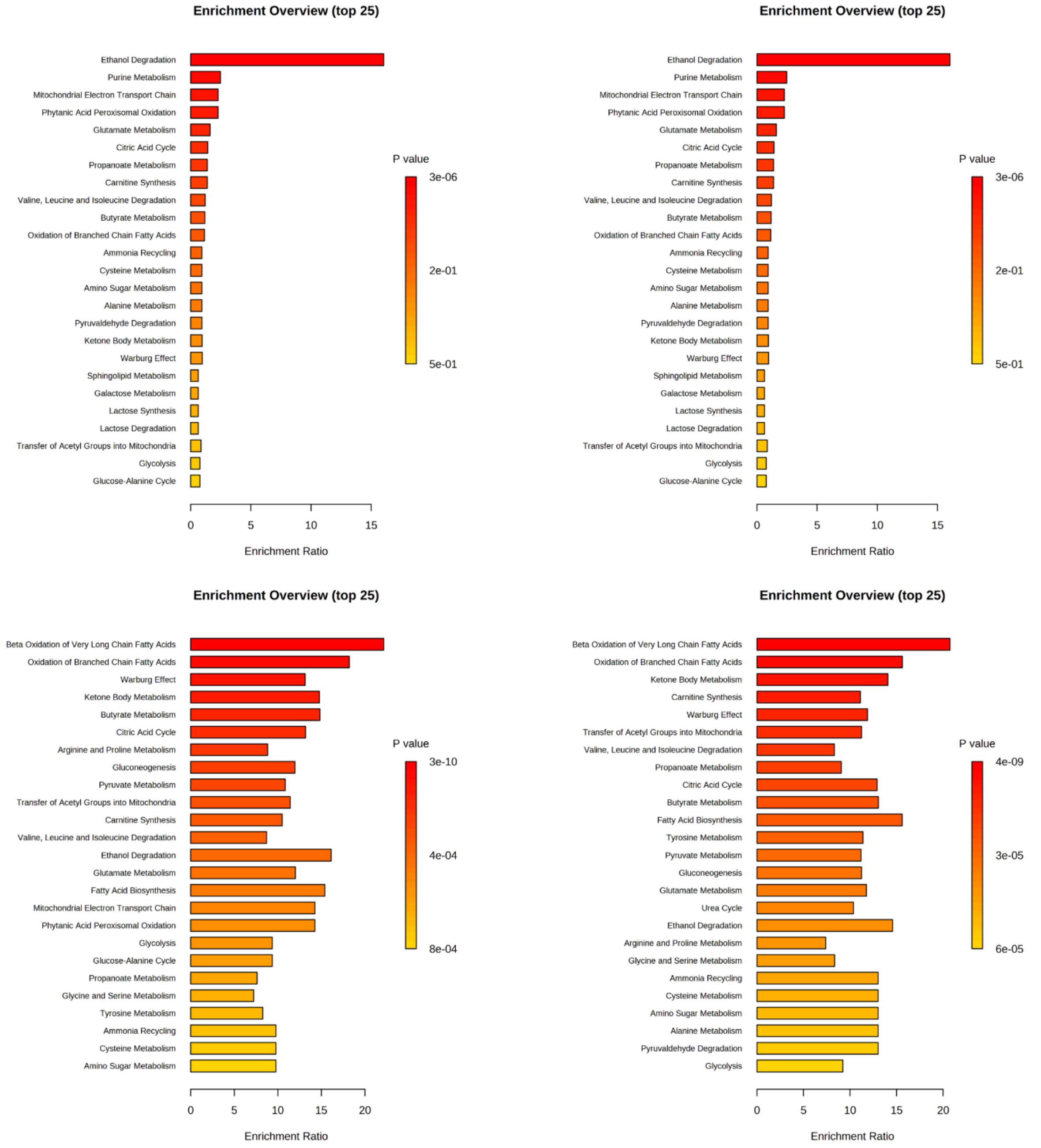

Figure S2: Quantitative enrichment analyses results showing top 25 metabolic pathways identified using the Small Molecule Pathway Database as reference. Pre-marathon profiles were compared to **(A)** 24 h and **(B)** 48 h post-marathon profiles, and immediately post-marathon profiles were compared to **(C)** and 24 h and **(D)** 48 h post-marathon profiles.

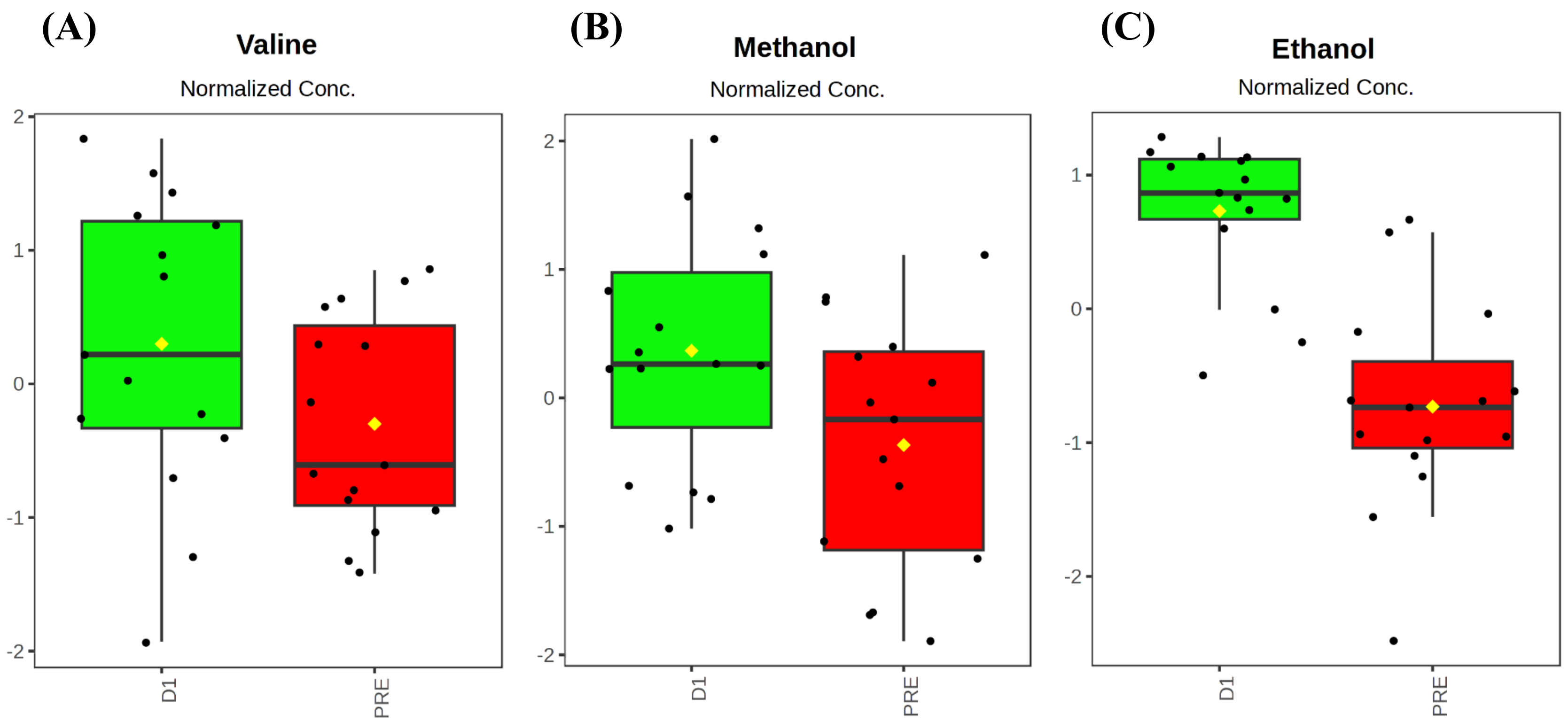

Figure S3: Boxplots indicating those metabolites that did not recover within 24 h post-marathon. Boxplots indicating baseline (PRE, red) metabolite concentrations relative to that at 24 h post-marathon (D1, green). Metabolites that did not recover include **(A)** valine, **(B)** methanol, and **(C)** ethanol.

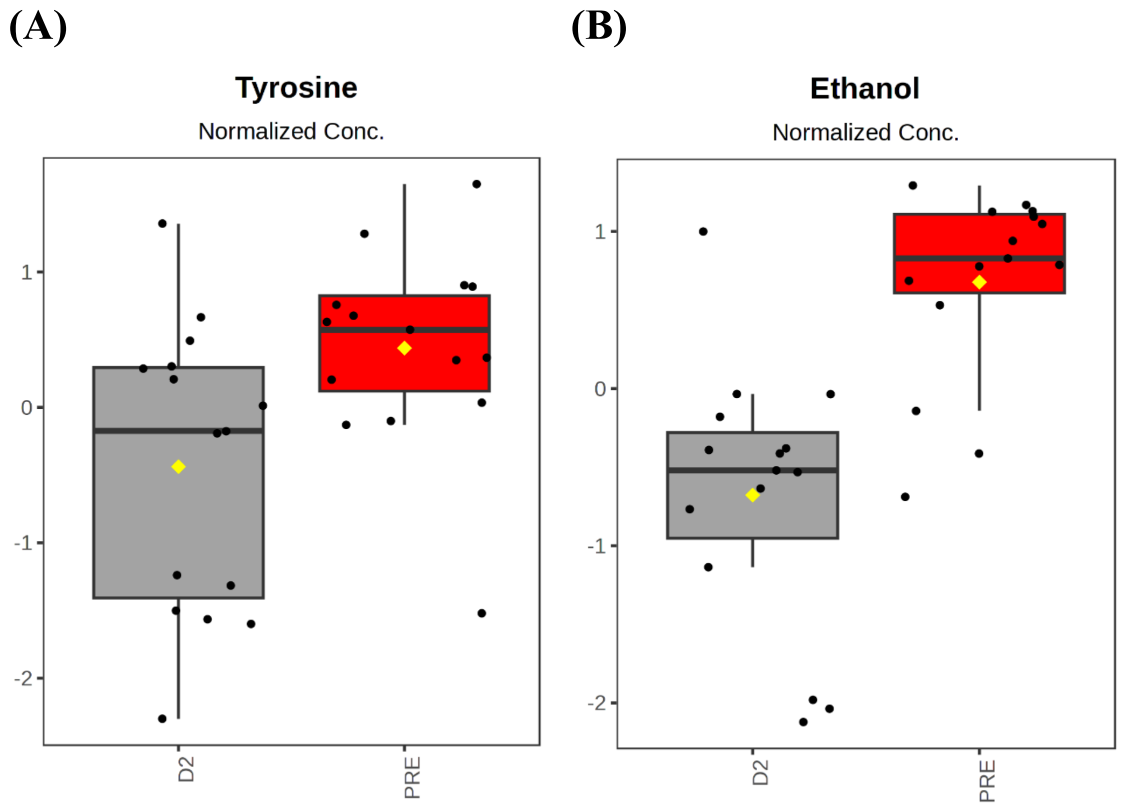

Figure S4: Boxplots indicating those metabolites that did not recover within 48 h post-marathon. Boxplots indicating baseline (PRE, red) metabolite concentrations relative to that at 48 h post-marathon (D2, grey). Metabolites that did not recover include **(A)** tyrosine and **(B)** ethanol.
